# Same emotion, different stimuli: A context-sensitive method to evoke nostalgia

**DOI:** 10.3758/s13428-026-02971-9

**Published:** 2026-04-07

**Authors:** Hetvi S. Doshi, Adam K. Anderson, Marlen Z. Gonzalez

**Affiliations:** Department of Psychology, Cornell University, Human Ecology Building 163, 37, Forest Home Dr., Ithaca, NY 14850 USA

**Keywords:** Emotion, Methods, Context, Nostalgia, Culture, Food, Idiographic, Nomothetic

## Abstract

Lived experience and current context are critical antecedents of emotions. However, practical methodologies for incorporating these factors into experimental emotion research remain scarce. We developed and tested an idiographic-nomothetic method to evoke a prototypically situated emotion: nostalgia. We describe the iterative qualitative and quantitative method, which involved (1) choosing cross-cultural target populations to manipulate present versus developmental context, (2) creating a conceptually and visually standardized food image set, (3) testing the nomothetic and idiographic factors in nostalgia, (4) measuring the outcomes’ replicability, and (5) testing the relation of state to global trait nostalgia. The method was examined on US university students who share the same current environment but originate from two distinct developmental contexts (USA vs. India). Across the two cohorts, nostalgic value exhibited an idiographically nomothetic pattern: vast variances between individuals coexisted alongside a cross-cultural pattern of high nostalgia for developmentally consistent foods within a person, which was greater for the culture further displaced from home. Nostalgia for food was consistent across time; high test–retest reliability was on par with food familiarity. Global trait nostalgia contributed to the propensity and intensity of evoked state nostalgia. We discuss this method’s usefulness for bridging phenomenological and positivist accounts of emotions while providing a practical way to systematically probe the impact of lived experience and context on their expression.

**Supplementary Information:**

The online version contains supplementary material available at 10.3758/s13428-026-02971-9.

## Introduction

The past is not dead. It’s not even past. - William Faulkner

The psychological study of emotions has been ground zero for contemporary debates on the importance of incorporating context into psychological science (Barrett, 2006; Cikara et al., 2022). Researchers argue whether experimental methods can consistently evoke emotions universally, whether labeled as anger, fear, disgust, or joy, without incorporating participants' unique life experiences. However, methods on how to incorporate contextual and idiographic factors remain scarce or prohibitive in time, if not money. Therefore, we created an iterative qualitative and quantitative method to probe the contextual, idiographic, and nomothetic factors of an uncontroversially *situated emotion*—nostalgia as cued by food. We follow the traditions of behavioral ecology (Gonzalez & Rice, 2024) and movements in psychology that embrace heterogeneity (Bryan et al., 2021) and the incorporation of context in theory and practice without giving up the goals of generalizability (Thomas, 2023). We offer a new standardized protocol by which sample and individual-specific emotions can be generated from the ground up. This method is rooted in the divergent experiences of individuals from different cultures who are occupying the same current environment. We further illustrate the method’s utility through two empirical studies probing idiographic and nomothetic factors.

### Nostalgia as a complex emotion

Emotions are a constellation of features, involving subjective experiences, physiological reactions, cognitive appraisals, behavioral tendencies, and regulation of thought, memory, and perception (Gross, 2015; Kreibig, 2010; Lerner et al., 2015; Scherer, 2005; Smith & Kirby, 2012). When designing methods that can account for how lived experience shapes emotional elicitation, focusing on a specific, well-defined case provides conceptual clarity. We use nostalgia as that case. Though studied less frequently than fear or happiness, nostalgia is uniquely representative of context-sensitive emotions. Rather than basic affect for sensory stimuli, nostalgia is felt in the present but oriented toward the past. It is most often evoked when people, places, or experiences once held dear are absent. Despite its unique specificity to one’s lived experience, the experience of nostalgia appears commonplace. Nostalgia is widely documented across time and culture—from ancient Greek accounts of *pothos* (Hillman, 1975), to Hofer’s seventeenth-century framing of nostalgia as homesickness (Hofer, 1688). Despite its association with longing and loss, empirical work largely situates nostalgia within positive psychology, linking it to self-continuity, social connection, emotional resilience, and meaning-making (Hepper & Dennis, 2023; Ismail et al., 2020; Layous et al., 2022). It buffers social threats (Sedikides et al., 2015a, b), enhances goal pursuit (Wildschut & Sedikides, 2023), and has clear behavioral consequences: from consumer preferences (Fan et al., 2020) to political alignment (Jacobsen, 2023). Indeed, corporations capitalize on nostalgia to influence purchasing behaviors across markets (Chrostowska, 2010). From children's cereal to retro tourism, nostalgia is used for emotional resonance and to drive purchasing decisions (Cho, 2023; Wang, 2023; Wulf et al., 2020). More critically, historians and social scientists posit that nostalgia is politically consequential. Idealized narratives of a “better past” often mobilize collective identity and belonging in ways that can lend affective weight to regressive or exclusionary ideologies (Bonikowski & Stuhler, 2022; Elgenius & Rydgren, 2022; Gest et al., 2017; Smeekes et al., 2021). The profound impact of nostalgia at the personal and social level highlights the need to deepen the methodological approaches to its study. Due to its situated nature—dependence on time and place—its study also advances our theoretical and empirical inquiry into the context-specific study of all emotions.

### The situated nature of nostalgia

Unlike with other emotions, nostalgia is presumed to be inherently autobiographical and context-specific (Salmose, 2019). It is prototypically experienced as a bittersweet emotion evoked by the reminder of something that cannot be accessed, except through memory, often triggered involuntarily by sensory or symbolic cues (Hepper et al., 2012; Ikeda & Kusumi, 2023). These memories tend to cluster around adolescence and early adulthood (Rubin & Schulkind, 1997), but what prompts them varies widely across cultural and developmental contexts. For example, food is a highly salient catalyst for nostalgia—both biologically and socially grounded—conjuring memories of belonging, identity, and care (Duruz, 1999; Green et al., 2023; Li et al., 2021; Mandal et al., 2022; Narchi et al., 2008; Sutton & Vournelis, 2009; Viladrich & Tagliaferro, 2016; Zhou et al., 2019). Important for methods attempting to induce nostalgia, food predicts heightened general and object-specific state nostalgia (Reid et al., 2023). Food also exemplifies how individual experience can carry shared cultural resonance. Transylvanian Jews recall prewar culinary life (Vasvári, 2021); Mexican migrants in California evoke home through ethnic grocery stores (Vázquez-Medina & Medina, 2015). Childhood objects or media may likewise evoke nostalgia without requiring spatial displacement, only temporal distance (Baxter, 2016; Lee & Hood, 2021). These examples underscore food nostalgia’s ability to emerge from personal memory while drawing on sociocultural symbols with collective significance.

### The measure of nostalgia in psychology

Scholars operationalize nostalgia either as a dispositional propensity to experience nostalgia—trait nostalgia—or as a distinct event spontaneously experienced—state nostalgia. Trait nostalgia is typically measured using survey scales. For example, the Southampton Nostalgia Scale (SNS) is a seven-item scale that assesses both the frequency of nostalgic reverie and the significance of nostalgia to the participant (Routledge et al., 2008). Trait nostalgia assessments like the SNS and others have a strong qualitative component: participants volunteer their perspectives on meaningfulness and characteristics of nostalgic reverie as well as unique nostalgic triggers and experiences. Studies using this scale have shown that dispositional proneness to nostalgia correlates positively with meaning in life (Sedikides & Wildschut, 2018) and with self-discontinuity caused by negative life events (Sedikides et al., 2015a, b). Presumably, those with higher trait nostalgia should be easier to induce into a state nostalgic experience. However, because nostalgia is context-dependent—it is felt towards objects and persons not easily available in the current spatial or temporal context—developmental experiences and displacement from that context should more strongly impact the proneness towards and intensity of nostalgic experiences over trait nostalgia.

Given that nostalgia is an interaction between the context, the person, and stimulus triggers, researchers cannot easily rely only on stimulus characteristics (e.g., a fearful face) to evoke nostalgia. Researchers induce nostalgia through writing exercises recounting autobiographical narratives (Abeyta et al., 2024; Kersten et al., 2020; Turner et al., 2022) or using evocative stimuli such as music (Mahon & Roth, 2023), smell (Reid et al., 2015), and food names/items (Reid et al., 2023). Sensory stimuli (e.g., sound, taste, smell) are the most frequent triggers of state nostalgia (Hepper et al., 2024), and in empirical settings they vary in the degree of participant specificity, ranging from small standardized databases of preselected stimuli (Janata et al., 2007) asserted as “likely to produce nostalgia” to unique participant-selected stimuli that are not shared across individuals (Asghar, 2018). Fully participant-selected stimuli allow for rich individualization but make it challenging to control for the role of third variables, such as low-level perceptual or high-level conceptual and semantic features. Standardized databases and protocols prioritize high interrater agreement, seeking to “on average” elicit nostalgia. However, this standardization introduces key limitations. For example, food image databases often strip contextual details bleached background, sterilized lighting, matched plating (Blechert et al., 2014; Foroni et al., 2013)—or present food in ways that are either overly appetizing or unrealistically artificial (Miccoli et al., 2014). Further, the reliance on interrater agreement, a strength in other research, can be a liability for studying nostalgia. Instead of pursuing generalizations across individuals, which sterilize context, we emphasize generalizing *within* individuals over time—intra-rater reliability—with the postulation that the nostalgic value of triggers is temporally stable. For this, we require high test–retest reliability on the items that evoke nostalgia, but low interrater reliability, highlighting both the individual stability and event variability underlying nostalgia.

### Balancing nomothetic and idiographic approaches

Taken as a whole, nostalgia is a highly complex emotion characterized by ambivalent valence, often situated in developmental experiences, but triggered by highly individualistic (idiographic) yet culturally meaningful (nomothetic) tokens and experiences of loss. It is no wonder, then, that nostalgia has found itself amidst contention between phenomenologists and positivists (Geniusas, 2025; Sedikides & Wildschut, 2024), whose views on everything from ontogenetic assumptions to methods of inquiry to generalizability are diametrically opposed. A similar need to balance nomothetic and idiographic concerns in conceptualization and measurement can be found in the study of psychopathology (Wright et al., 2019). The *nomothetic approach* emphasizes laws (*what always is*) and is central to experimental psychology. In practice, this emphasis often involves reducing intergroup variability using group averages and statistical significance to draw broad conclusions (Barlow & Nock, 2009; Hurtado-Parrado & López-López, 2015). In contrast, the *idiographic approach* prioritizes lived experience (*what once was*), treating the person as the primary unit of analysis (De Luca Picione, 2015; Lamiell, 1998), and is traditionally emphasized in clinical case studies. Some researchers have moved towards an “idiographically nomothetic” model of psychopathology, recognizing the many unique experiences and yet shared struggles of individuals diagnosed with the same disorder (Beltz et al., 2016). With the benefit of many available measures and a long history in the study of psychopathology, researchers have focused on statistical innovations to model how person-specific maps give rise to group-level structure (Gates & Molenaar, 2012).

While nostalgia studies benefit from both idiographic and nomothetic methods for inducing nostalgia (for an overview, see Wildschut & Sedikides, 2024), they are missing a method that balances granular details of an individual’s experience and generalizability. Our work towards an “idiographically nomothetic” approach to food nostalgia begins with establishing a testing protocol to build population-specific stimuli and a research protocol to derive both idiographic and nomothetic information systematically. We take an approach more akin to behavioral ecologists. We focus on how ontogenetic factors, across two distinct cultures living at the present moment in the same place, impact idiographic and nomothetic experiences of state nostalgia. As reviewed above, experiences in childhood and adolescence tend to be focal to nostalgic experiences. Displacement in time and space also increases the likelihood of nostalgia. Therefore, we explore a method that capitalizes on the experiences of displacement within the neighborhood-like university context (Tan et al., 2024) to capture both individual- and group-level variance in state nostalgia systematically while embracing heterogeneity as a signal.

### The present study

We developed an idiographic-nomothetic approach to evoke a situated emotion—nostalgia for food—in students currently experiencing the same environment (a US university) but coming from two distinct developmental contexts (US Americans raised in the USA vs. Indians raised in India). This method afforded a manipulation and examination of one’s developmental cultural context, and current displacement from it, on nostalgia. We engaged in an iterative qualitative and quantitative process, starting with choosing target populations for whom we created an ecological, visually standardized cross-cultural food image set, to testing the nomothetic and idiographic factors in food-related nostalgia in our samples. For our empirical studies, we hypothesized a combination of nomothetic and idiographic effects using our normed image set. Nomothetic hypotheses were that (1) groups would have greater nostalgia for culturally consistent foods, and (2) consistent with their displacement, Indian participants would express higher nostalgia than their US peers. Idiographic hypotheses posited that (1) images would show poor interrater reliability with participants having their own unique nostalgia profiles, and (2) images would have high test–retest reliability within the individual. Further, we use this method to examine the role of trait nostalgia and the magnitude of its contribution to food-evoked nostalgia. We discuss how the results highlight the usefulness of our process to bridge phenomenological and positivist accounts of nostalgia while providing a practical way to systematically probe the impact of lived experience and context on emotional experience.

## Method

### Participants

We recruited two cohorts from the undergraduate and graduate student populations (Cohort 1: *N* = 46; 21 Indians, 25 US Americans; Cohort 2: *N* = 52; 26 Indians, 26 US Americans). Combined, the study had 98 participants: 51 participants (7 male) were from the USA, between 18 and 30 years of age (*M =* 20.41, *SD =* 1.97), and 47 participants (24 male) were from India (born and raised in India until at least age 15 years currently living in the USA), aged 22 to 62 (*M =* 27.36, *SD =* 8.32). The racial composition of the US American group self-identified as 10% African American, 6% Latino, 33% Asian (non-Indian), 39% Caucasian, and 12% Mixed. Data from Cohort 1 were collected from February to June 2022. A subset of Cohort 1 (*n* = 33) returned at least 60 days (*M =* 113.48*, SD =* 32.04*)* after participation to repeat the study from July to October 2022. Data from Cohort 2 were collected from October 2022 to November 2023. The sample size of all cohorts was a priori confirmed to be significant in power (Appendix A). Participants received either course credit or nominal monetary compensation ($8/h). For cohort-wise demographic breakdown, refer to Appendix B. Cornell University’s Institutional Review Board reviewed all methods.

### Population selection

Participants were prescreened via an online survey to assess eligibility. To control for cultural context, only participants born and raised in either India or the USA were included. Indian Americans were excluded to avoid confounds. To control for disordered relationships with food, we assessed and excluded individuals with eating disorder symptoms or history of a diagnosis via the Eating Disorder Diagnostic Scale (EDDS; Stice et al., 2000; see measures). Individuals who passed the screening were informed of their eligibility, learned the study procedure, provided digital informed consent if interested, and were scheduled for an in-lab session.

### Conceptualization

**Fig. 1:**
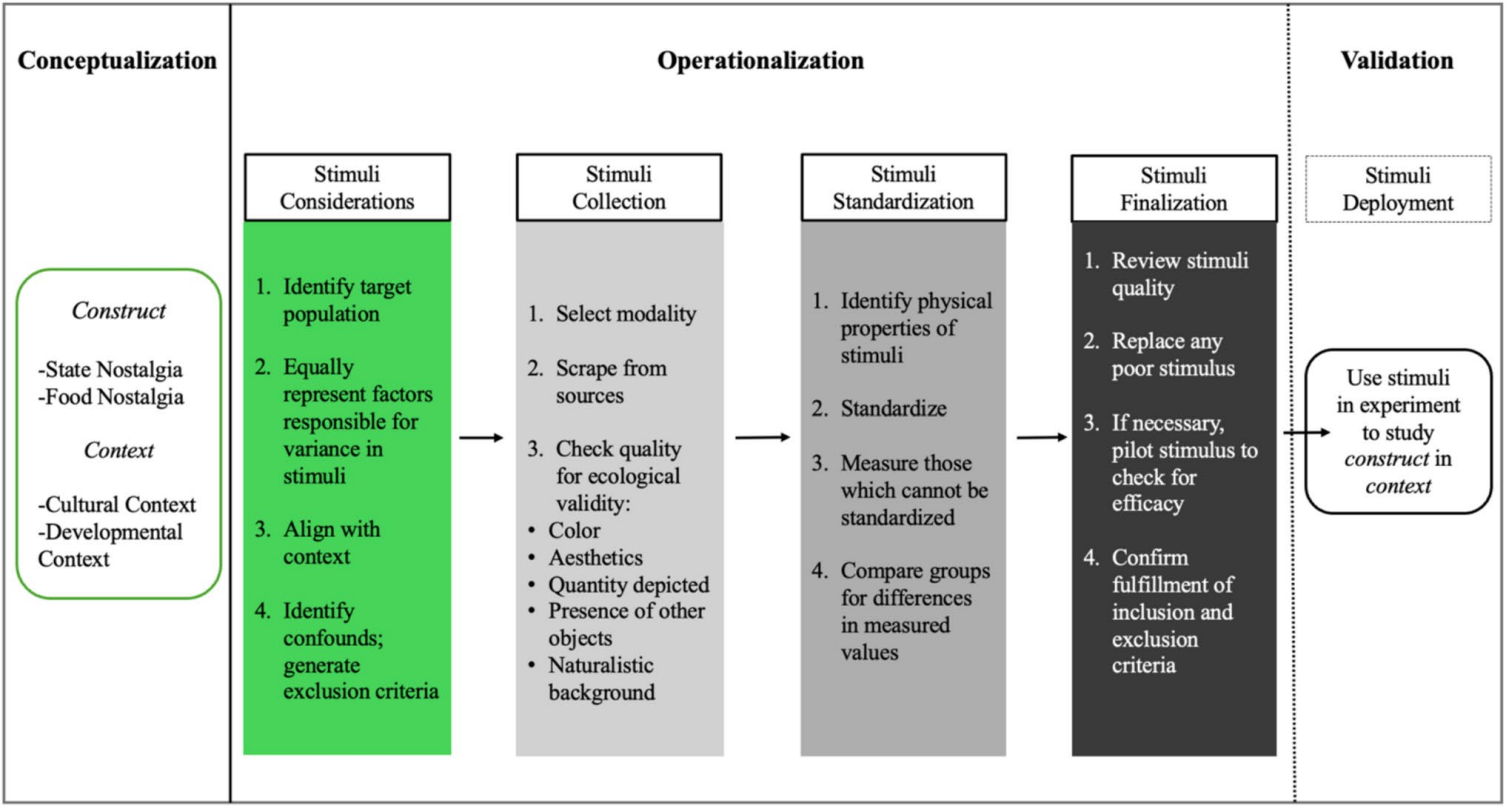
Overview of the method to build context-sensitive stimuli

Figure 1 provides an overview of the general steps in our method. At the conceptualization stage, we first considered our target context and chose Indian and US American participants within the university context. This target was ideal because the groups shared current experiences (e.g., life stage, leaving home, similar goals) but differed substantially in the culture they were raised in. Other considerations were the location of the research, permitting easier recruitment, and the cultural competence of the research team, permitting easier stimuli search. The next steps concerned the creation of a culturally sensitive and evocative stimulus set that was visually normed. Finally, we validated our conceptual and measurement choices via two empirical studies.

### Operationalization

#### Stimuli considerations

Stimuli consideration involved an iterative process of expansively generating culturally relevant foods and then qualitatively refining the list. The research team (half Indian and half US American) created a list of food items typically consumed in both countries. Items on the list were collected through oral surveys of friends and colleagues, online searches for common foods, popular food websites, validated studies, pilot data, and hypotheses. Qualitative refinement involved examining the heterogeneity of our food list towards creating a representational and generalizable stimuli set. We sought to maximize potential familiarity, consumption frequency, and emotional and social value for a large audience, and minimize brand biases and differences in psycho-hedonic responses to sweet versus savory tastes (Lim et al., 2020). We excluded raw foods, branded foods (with a few exceptions listed in Supplement 1), drinks, and sweet items. We considered ingredients, calories (Appendix C), portion sizes, cuisines, subcultures, regions of origin and consumption, popularity, time (breakfast to festivals), and place of consumption (home to restaurant). We created a list of roughly 220 items that wholly covered both countries and were approximately evenly distributed across these factors (Supplement 1). This list was further screened by two research assistants of the same age and from the target cultures of our population. Research assistants indicated for each food item from their culture whether it was familiar, likable, and available during their development (1 = yes, 0 = no) (Supplement 2). The research team then reviewed the item ratings to support further qualitative decisions on creating an inclusive but not esoteric food list.

#### Stimuli collection

After we developed a list of culturally common foods, we chose to collect visual stimuli representative of these items. While words can serve as retrieval cues for autobiographical experiences related to nostalgia (Reid et al., 2023), visual images elicit robust psychological and physiological responses similar to real food presentation (Avery et al., 2021). We sourced our stimuli from publicly available images—online recipes, Google Images, online public posts—and subsequently requested permission to use the images. The research team adopted a simultaneous recursive process where multiple research assistants would search for a set of items until they found one that met our image criteria standards (Box 1). In most cases, only one image was considered acceptable. However, 24 items had two acceptable images, and one item had three acceptable images. These were viewed only by Cohort 1.

Food items whose image acquisition was difficult (due to rare or poor quality of images) were replaced with a new food item.

**Box 1** Ecological and cognitive criteria for image selection.

**Table:** 

1. An image must be in color.
2. An image must have a background that is also in color.
3. An image should be minimal in its aestheticism.
4. An image should be realistic in the portion sizes depicted.
5. The food item must be in the center of the image.
6. An image must have minimal other foods in the background.
7. The principal item in the image must not be unnaturally cropped nor pixelated upon resizing.

#### Stimuli standardization

Visual features of the selected images were further normalized and measured following best practices (Knebel et al., 2008), because salient visual features can alter eye movements, attention, memory, and decision-making (Niu et al., 2012). Two tools were used for standardization and measurement: ImageJ, a Java-based image processing program (Pérez & Pascau, 2013), and the SHINE toolbox from MATLAB (Willenbockel et al., 2010). Group-level evaluations were conducted using RStudio. All raw values of physical properties, scripts for standardization, measurement, and comparison, and results and graphs can be found on our GitHub repository [https://github.com/hetvidoshi/contextsensitivestimulimethod.git].

##### Normalization

All images were resized (cropped/expanded and not warped) to 500 × 500 pixels (squares). Then, brightness—i.e., luminance—and contrast were calibrated using a linear histogram stretch process, and contrast was enhanced with a saturation of 0.35, as per the default setting of ImageJ’s normalization process (Ferreira & Rasband, 2011; Klemm & Miura, 2022).

##### Measurement

We measured the texture, luminance, saturation, hue, and mean gray values (grayscale or mean RGB values) of each image. We then compared images of Indian and US foods to assess for group-level differences in physical properties.

#### Stimuli finalization

Cohort 1 viewed 228 images (134 Indian, 95 US foods) for both timepoints, and Cohort 2 viewed 217 images (102 Indian, 115 US foods). Only data from the 194 images viewed by all participants are reported in this paper. Further information is provided in Appendix D. The stimuli set can be found on our GitHub repository [https://github.com/hetvidoshi/contextsensitivestimulimethod.git].

### Validation

#### Measures

Screening measure: The EDDS is a 22-item self-report measure for diagnosing anorexia nervosa (e.g., “Have you felt fat?”), bulimia nervosa (e.g., “How many times per week on average over the past 3 months have you made yourself vomit to prevent weight gain or counteract the effects of eating?”), and binge-eating disorder (e.g., “During these episodes of overeating did you eat large amounts of food when you didn’t feel physically hungry?”) (Stice et al., 2000). The scoring algorithm created by Stice et al. (2000) was used to diagnose each person for all three disorders. The eligibility calculation script, with the algorithm, can be found on our GitHub [https://github.com/hetvidoshi/contextsensitivestimulimethod.git]. The scale was previously validated with a Cronbach’s alpha of .91.

Global trait nostalgia was measured using the Southampton Nostalgia Scale (SNS) (Sedikides et al., 2008). The SNS is a seven-item scale measuring trait nostalgia. Four items measure propensity to nostalgize or frequency of nostalgizing (e.g., “How often do you experience nostalgia?”), and the remaining three measure the value of nostalgia (e.g., “How important is it for you to bring to mind nostalgic experiences?”). A high score on the seven items indicates high trait nostalgia, in particular proneness to experiencing nostalgia. Cronbach’s alpha for a previous study was .93 (Barrett et al., 2010). This trait measure was added for Cohort 2.

Item-level familiarity, comfort, and nostalgic value were assessed through singular questions on a seven-point Likert scale (1 = Not at all; 7 = Extremely). Familiarity was assessed with the question *“How familiar is this food item to you?”* (Colla et al., 2021). Comfort was assessed with the question “*Do you consider this food item to be a comfort food?”.* Nostalgic value was assessed with the question *“Does this food hold any nostalgic value for you?”.* Additionally, hunger was measured before and after the experiment as a manipulation check with the question “*How hungry are you?*” on a 10-point Likert scale (1 = Not at all; 10 = Extremely) (Hollis, 2022).

#### State nostalgia proneness and magnitude

We created summary scores capturing trait-level properties of state nostalgia based on participant behavior during our study. We operationalized state nostalgia proneness as the frequency with which a participant rated items as having high nostalgic value, i.e., the number of items rated equal to or greater than 6 in nostalgic value. We operationalized an individual’s state nostalgia magnitude as the average intensity of the nostalgic value ratings, i.e., the average nostalgic value for all items.

#### Food-nostalgia and familiarity procedure

**Fig. 2:**
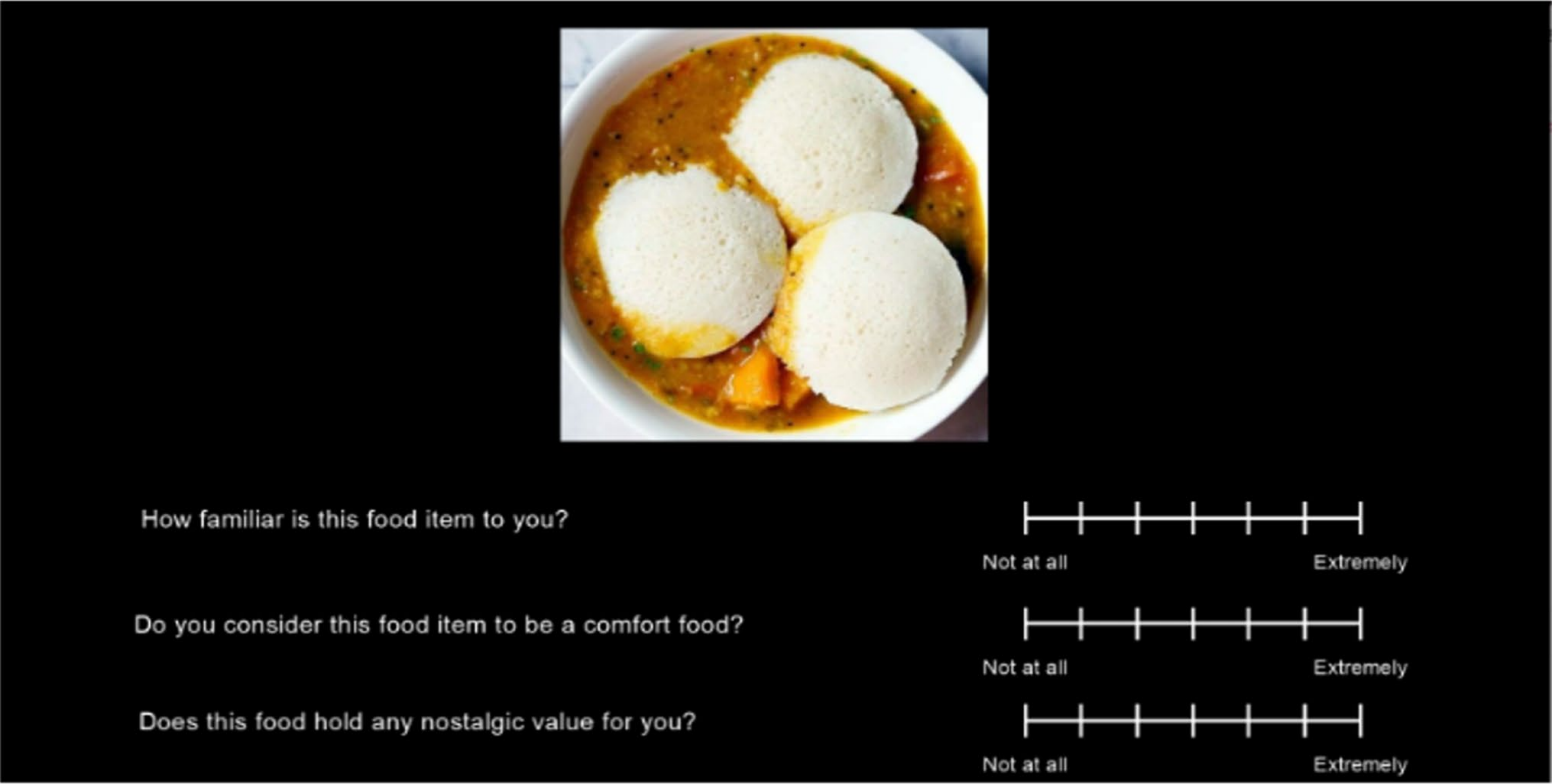
Experimental paradigm for rating the images. The example image is an Indian food item called *Idli* (white pieces) and *Sambhar* (the curry underneath), traditionally eaten together.

To assess the ability of our food images to evoke nostalgia, participants rated the level of nostalgic value for each image. Each participant was seated approximately 60 cm from the computer. The stimuli were presented on a computer using PsychoPy (Peirce, 2007). Images were presented in 24-bit color at a vertical and horizontal visual angle of 11.47°. Each participant rated their hunger before stimulus viewing. Participants then viewed each image in a random order as set by PsychoPy’s randomization module. Images were viewed in blocks of 20 with 15 second breaks. Each image was presented for 10 seconds along with three questions below the image measuring familiarity, comfort, and nostalgic value on a seven-point Likert scale (1 = Not at all, 7 = Extremely; Fig. 2). The order of questions was identical for all trials. An attention check--i.e., a space bar press required for a picture of a raw vegetable--appeared after the third, sixth, and ninth blocks. After the last image, i.e., task completion, participants rated their hunger again. Cohort 1 was invited to return 60 days later to complete the same process with a new randomized image order. The cohort of the first session will be referred to as “Cohort 1, timepoint 1” or “Cohort 1 T1.” The subset that returned will be subsequently referred to as “Cohort 1, timepoint 2” or “Cohort 1 T2.” Cohort 2 was a new set of participants who completed the same procedure, but with an additional component that was interspersed with the ratings task, in which they described autobiographical memories of selected food items. The comfort ratings for all cohorts and the additional component for Cohort 2 will be reported separately from this manuscript. For those items that had more than one acceptable image, the items that scored lower in nostalgic value were removed from further analysis and use.

### Data analysis and reporting strategy

We assessed test–retest reliability between timepoint 1 (T1) and timepoint 2 (T2) in the subset of Cohort 1 that returned (*n* = 33) using three analysis methods: Spearman correlation, linear mixed-effects modeling, and an equivalence test of two one-sided *t*-tests (TOST). The validity of the stimuli was established through both a test of convergent validity to trait nostalgia and divergent validity from familiarity using linear mixed-effects modeling. Nomothetic trends in nostalgic value were identified using both linear and logistic mixed-effects models, and idiographic patterns were isolated by measuring interrater reliability and visually mapping individual profiles.

All mixed-effects models were conditional with random intercepts only. The standardized metric reported for effect size is partial eta-squared (η_p_^2^) for simple and multiple linear regressions, and Cohen's *d* for *t*-tests. For the former, η_p_^2^ = .01 indicates a small effect, .06 a medium effect, and η_p_^2^ = .14 a large effect (Richardson, 2011). For error estimation reports, the 95% confidence interval is used for simple *t*-tests, and standard error for regression models (excluding the *F*-statistic). An alpha of .05 was used to reject the null hypothesis. Fixed and random effects for each model can be found next to its result, including any *z*-score normalization information. The intraclass correlation coefficients of all random effects adjusted for fixed effects in every model can be found in Appendix E.

All results other than test–retest reliability were derived from combining Cohort 1 T1 and Cohort 2. Binned nostalgic value in relevant analyses was derived from dividing the discrete seven-point scale into low (1–2), medium (3–5), and high (6–7). For all results, “home country” refers to the participant’s developmental history—India and USA—and “food country” is the country where these foods are commonly consumed—India and USA. Participants born and raised in the USA are referred to as “US Americans,” and those who grew up in India are “Indians.” “Food relationship” is a label of the participant’s developmental relationship to the food; for example, USA foods are labeled as “home” for US Americans and “foreign” for Indians. Variance at different predicted values was roughly equal for all models, suggesting that homogeneity of variance was achieved despite the unequal frequency of each level within home country and food relationship.

## Results

### Covariates and manipulation check

#### Assessment of visual features

A Welch two-sample unpaired *t*-test was used for comparing US and Indian food images on each of the five image properties measured. Hue and texture were not significantly different between the two groups—hue: *t*(227.66)* =* 0.27, *p = .*78, 95% CI [−0.02, 0.02], *d =* 0.04; texture: *t*(226.70)* =* −0.53, *p = .*60, 95% CI [−2.85, 1.65], *d =* −0.07. Luminance, saturation, and mean gray values were significantly different between US and Indian food images—luminance: *t*(219.62)* =* 5.23, *p* < .001, 95% CI [11.32, 24.98], *d =* 0.69; saturation: *t*(226.07)* =* −3.75, *p* < .001, 95% CI [−0.09, −0.02], *d =* −0.50; mean gray values: *t*(219.73)* =* 5.56, *p* < .001, 95% CI [11.99, 25.17], *d =* 0.73. To test whether these properties were associated with nostalgic value ratings, we ran separate linear mixed-effects models with *z*-scored luminance, mean gray values, and saturation as fixed effects, and participant and image as random effects. Saturation showed a small association with nostalgic value, *t*(310.39)* =* 2.16, *p = .*03, η_p_^2^* = *.01, but luminance and mean gray values did not—luminance: *t*(479.39)* =* −0.16, *p = .*87, η_p_^2^ = .00; mean gray values: *t*(449.97)* =* −0.06, *p = .*95, η_p_^2^ = .00. On further examination, the distribution of saturation values was similar between images in the two food groups. As a precaution, saturation was added as a covariate in models of nostalgic value that included food relationship.

#### Hunger manipulation check

We evaluated whether the images were evocative of real food by testing pre- and post-experiment hunger ratings. On average, hunger increased by 2.2 points (22%) for members of Cohort 1 at timepoint 1 (*SD* = 2.1) and 1.3 points (13%) at timepoint 2 (*SD =* 2.4), and 1.8 points (18%) for Cohort 2 (*SD* = 3.4). A paired pre- and post-hunger *t*-test of all participants in Cohorts 1 and 2 showed that participants were hungrier after completion of image viewing, *t*(96)* =* 6.94, *p* < .001, 95% CI [1.42, 2.55], *d =* 0.70. When comparing the two cohorts in their pre- and post-study hunger, we found no significant difference between the two cohorts in the magnitude of hunger increase, *F*(1, 95) = 0.42, *p = .*52, η_p_^2^ = .00, or between Indian and US participants in the aggregated sample, *F*(1, 95) = 1.14, *p* = .29, η_p_^2^ = .01. Across all samples, the magnitude of hunger difference was not associated with state nostalgia magnitude, *F*(1, 95) = 0.05, *p = .*83, η_p_^2^ = .00, or nostalgia proneness, *F*(1, 95) = 0.04, *p = *.84, η_p_^2^ = .00.

### Intra-individual test–retest reliability

#### Nostalgic value stable across timepoints within participants

**Fig. 3:**
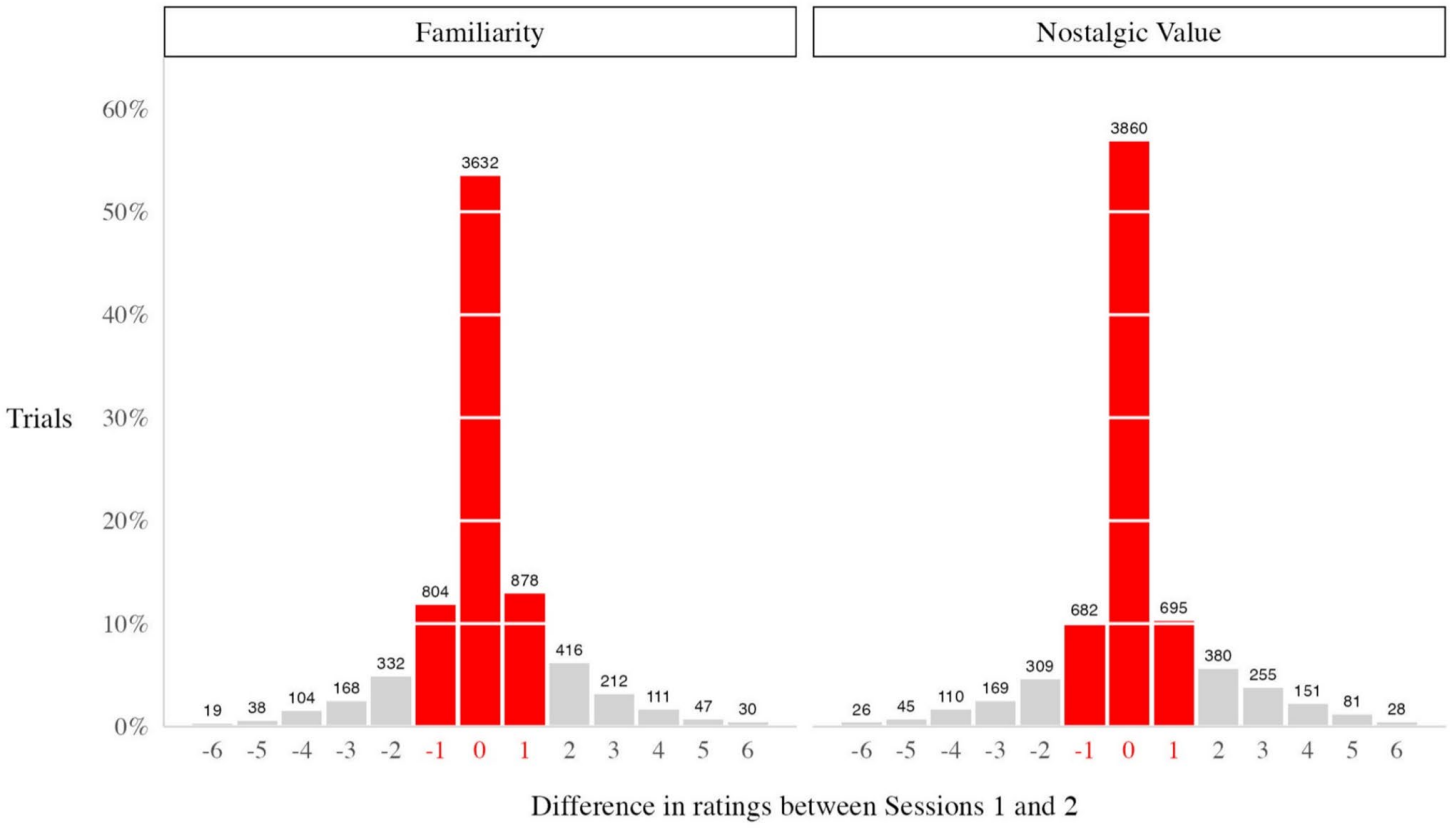
Number of trials at each difference value in ratings between Sessions 1 and 2 for familiarity and nostalgic value. Approximately 80% of the trials fall within ±1 for both variables, marked in red. Maximum difference is an absolute value of 6 because the Likert scale ranged from 1 to 7.

We assessed test–retest reliability in Cohort 1 across timepoints T1 and T2 (*n* = 33) using three analysis methods. For our first assessment using Spearman correlations, we found that item ratings (concatenated across participants) were significantly and similarly positively correlated between different timepoints for familiarity, *r*(6,580) = .76, *p < *.001, and nostalgic value, *r*(6,499) =.77, *p < *.001. No other variables moderated these correlations (Appendix F). In our second assessment using a linear mixed-effects model with participants and images as random effects, we see the same predictive value of T1 on T2 for familiarity, *F*(1, 4,611.4)* =* 6,874.5, *p < *.001, η_p_^2^ = .60, and nostalgic value, *F*(1, 5,519.7)* =* 9,157.4, *p < *.001, η_p_^2^ = .62. Finally, equivalence tests (TOST) were significant, confirming that the difference in ratings from T1 and T2 was within the predefined equivalence region—Δₗ = −1 and Δᵤ = 1—for both familiarity, with a mean difference of 0.07 (90% CI [0.01, 0.13], *p* < .001), and nostalgia, with a mean difference of 0.11 (90% CI [0.04, 0.18], *p* < .001). A total of 78.23% of familiarity trials and 77.10% of nostalgic value trials fell within *±*1 rating point on a scale of 1 to 7 (Fig. 3). Thus, state nostalgic value, however complex and subjective its individual origins from past experiences are, was as reliable as simple food familiarity. We will address the relationship between familiarity and nostalgia further below.

### Inter-individual heterogeneity

As predicted, we found strong homogeneity, i.e., consistency, in nostalgia food triggers over time within a person. We then evaluated our second prediction of substantial heterogeneity in what food items evoke nostalgia: food items that have nostalgic value are not reliable across people, even from the same cultural developmental context (see Table 1).

**Table 1:** Krippendorff’s *α* values for ratings of nostalgic value within groups within cohorts

Variable	Home country	Food relationship	Krippendorff’s α	
			Cohort 1	Cohort 2
Nostalgic value	India	Home	.17	.23
		Foreign	.16	.21
	USA	Home	.18	.16
		Foreign	.15	.15

#### Low interrater agreement on nostalgic value within home country and food relationship

We calculated interrater agreement for all stimuli, aggregated and within groups. Krippendorff’s alpha (α) (Krippendorff, 2011) was selected for agreement calculation because it works well with missing values (2.8% of the trials) and varying cohort sizes. An ordinal method, as appropriate for Likert ratings, was used to calculate alpha for participants and items within each group (disaggregated by cohort, home country, and food relationship). The test of homogeneity revealed high heterogeneity between participants in their ratings of nostalgic value: across all trials and both groups—Indians and US Americans—Krippendorff’s α was .09, which is close to zero. There was greater agreement within groups; Table 1 shows alpha values for disaggregated data. In contrast to the high test–retest reliability within individuals, there was high heterogeneity in nostalgic value for items when examined at the group level, both across cohorts and across home and foreign foods for each home country.

#### Individual-specific profiles of nostalgic value

**Fig. 4:**
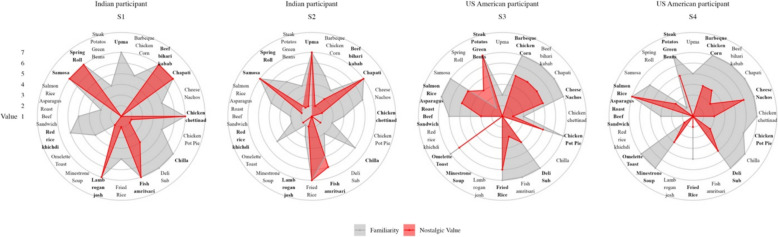
Two Indians’ and two US Americans’ ratings of familiarity (gray) and nostalgic value (red) for 10 home foods and 10 foreign foods. Boldface *x*-axis labels mark home foods, i.e., India foods for Indian subjects and USA foods for US American subjects

We pseudo-randomly selected 20 food items using R functions (refer to analyses script, section k) and created participant profiles to visualize the heterogeneity of nostalgic value, at the individual level, against baseline familiarity values. We showcase four individuals’ responses in Fig. 4 for illustrative purposes. The graph shows variation in nostalgic value for the same items, even for individuals from the same home country. For example, when rating nostalgic value for *spring roll,* a north Indian appetizer, one Indian subject (S1) gave it the highest value (7), whereas the other (S2) gave it the lowest (1). Similarly, when rating *omelette toast*, a US American breakfast, a US American subject’s (S3) nostalgic value was 6, whereas another (S4) rated it at 2. Familiarity with the foods was not sufficient for nostalgia, as shown by the inconsistent overlap of colors. Despite familiarity with foreign foods, individuals found more of their home foods to be nostalgic, though there were also some exceptions to this overall pattern. Individuals differed substantially in their propensity to feel nostalgia for these 20 foods, which is exemplified in the comparison between S3 (higher ratings overall) and S4 (lower ratings overall).

### Idiographic nostalgic values at the individual level

#### Participant pool showed variability in images’ nostalgic value

**Fig. 5:**
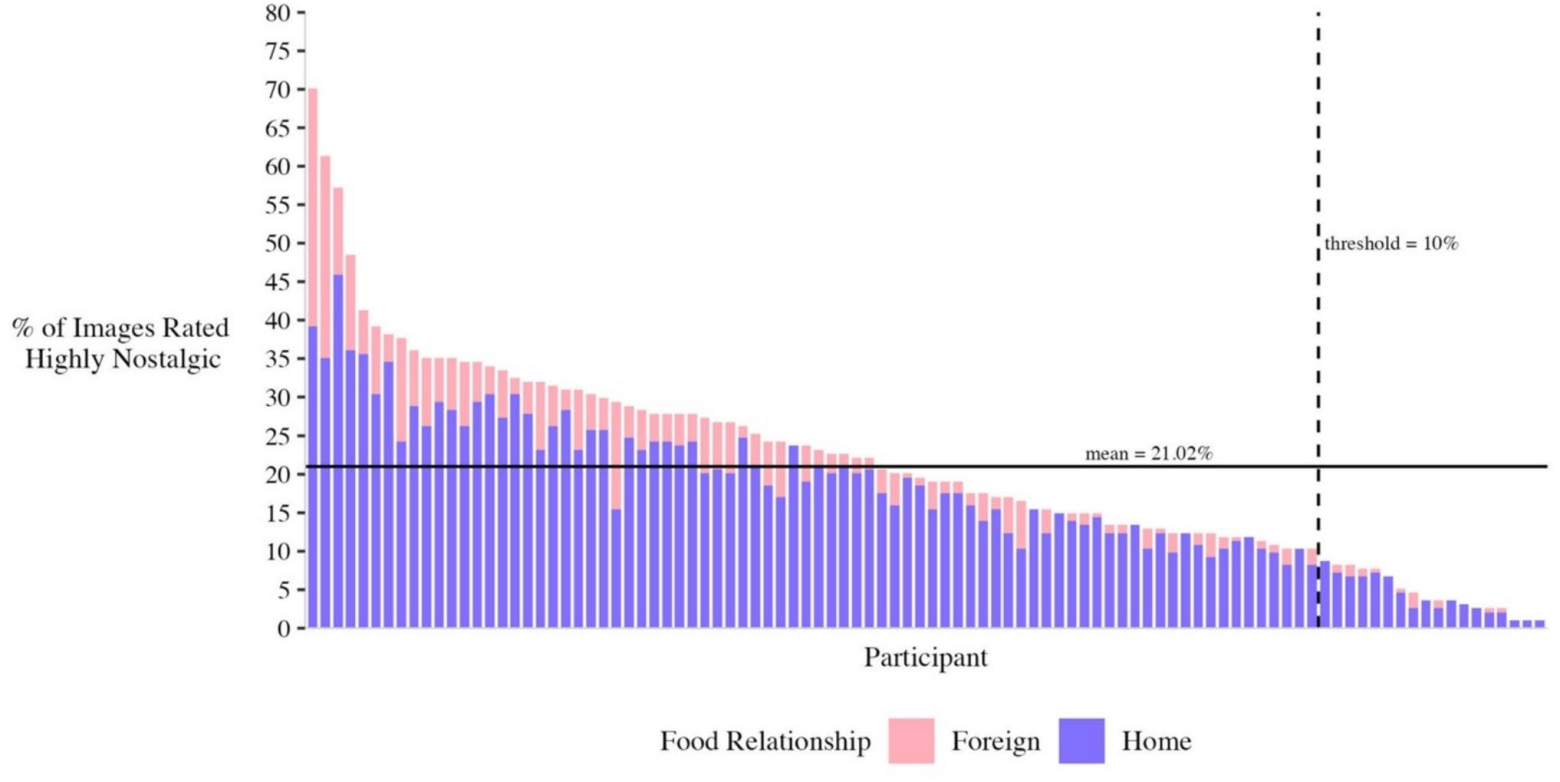
Stacked bar chart of the percentage of images rated high in nostalgic value by each participant, split by food relationship and rank ordered by participants’ nostalgia propensity (home foods in purple, foreign foods in pink). A solid horizontal line marks the mean intercept. A dashed vertical line marks a threshold at 10% of the stimuli

For any given individual, an average of 21.02% (40.78) images were rated as evoking high levels of nostalgia (*SD* = 13.25%) (Fig. 5). Individuals ranged in the percentage of images rated as highly nostalgic from as little as 1.01% to as much as 70.10% (mean absolute deviation = 12.99%), with 81.6% of all participants finding at least 10% of the stimuli (approximately 19.4 images) to be highly nostalgic. Individuals differed substantially in the frequency of high nostalgia responses to images. Home country differences are described below.

#### Items consistently ranked as having high nostalgic value

**Fig. 6:**
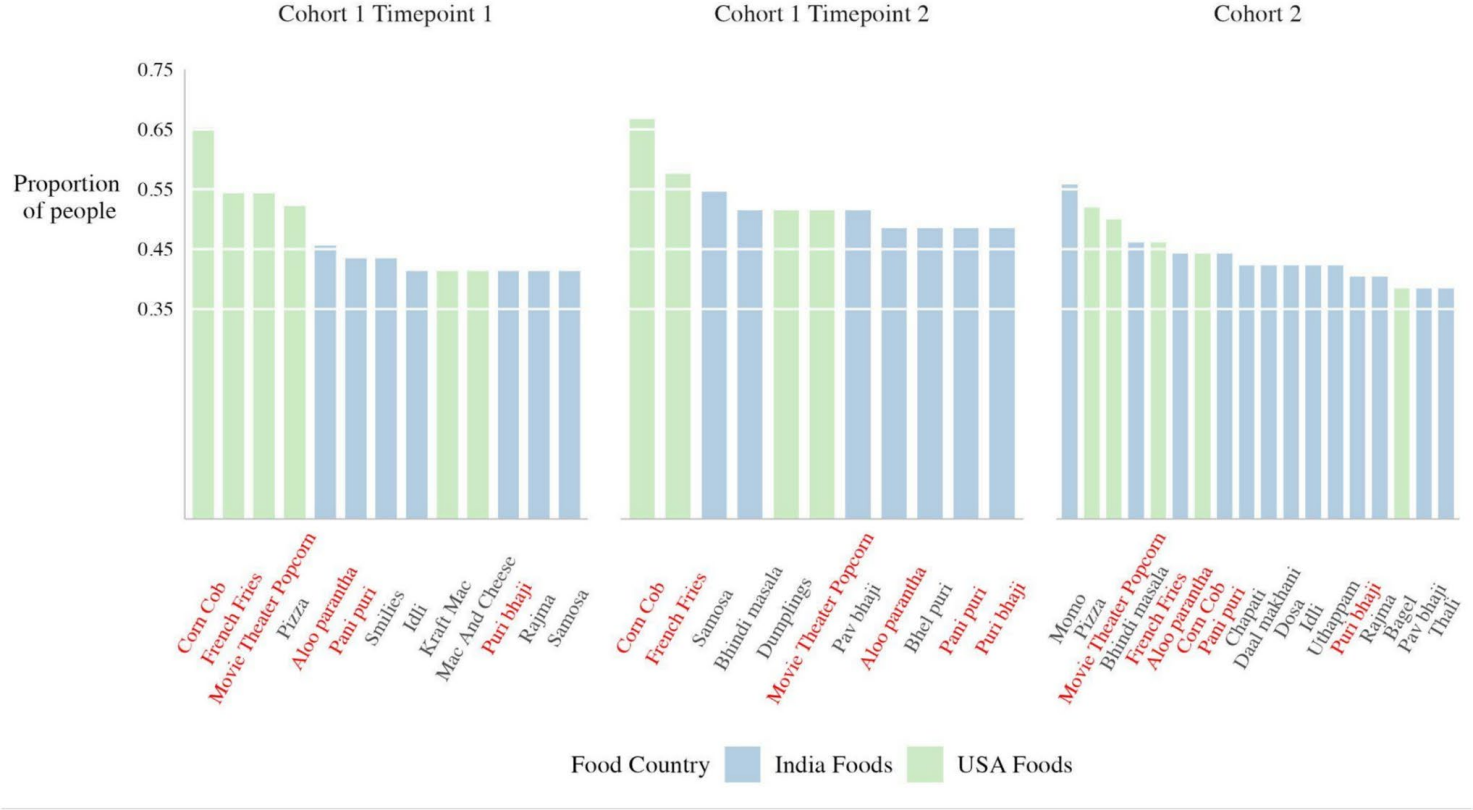
Food items that were most frequently valued as highly nostalgic by individuals of a cohort, collapsed across home country. Bar color marks the food’s country association (Indian foods in blue, US foods in green)*.* Items in red were consistently rated as highly nostalgic across cohorts

To see whether certain items had consistently high nostalgic valuations such that they could be used nomothetically across individuals, we extracted items for each cohort whose proportion of high nostalgic value ratings was at or above 1.5 standard deviations from the mean proportion of high nostalgic value ratings. For Cohort 1, timepoint 1, 13 items were agreed to be highly nostalgic, meeting the 1.5 standard deviation criteria. For Cohort 1, timepoint 2, 11 items met the criteria. Of these, seven items were repeats from timepoint 1. In Cohort 2, 18 items met the criteria. Of these, nine were the same as in Cohort 1, timepoint 1. Six items met the criteria across all cohorts: *corn on the cob, movie theater popcorn, French fries, aloo paratha, pani puri,* and *puri bhaji* (Fig. 6). Only 3% of items were consistently evocative of high nostalgia, with a maximum agreement of 66.67% participants for the top item (corn on the cob). Few foods consistently evoked high nostalgia across samples.

### Nomothetic nostalgic values based on food relationship

We examined the effect of food relationship on nostalgic value in our nomothetic investigations, as warranted by our joint idiographic-nomothetic framework and our hypothesis on the influence of the developmental context on nostalgia later in life.

#### Home foods were much more likely than foreign foods to be rated as high in nostalgic value

A smaller percentage of foreign foods (*M =* 8.49%, *SD* = 8.77) than home foods (*M =* 36.17%, *SD* = 22.00) were labeled as high in nostalgic value, receiving a rating of 6–7 (Table 2). In general, home foods had higher nostalgic value, *t*(274.7)* =* 80.11, *p < *.001, η_p_^2^ = .26, collapsed across all items. We next assessed the probability of low, medium, or high nostalgic value ratings given food relationship. We used binned nostalgic values to estimate the odds of belonging to a bin with two logistic mixed-effects models with food relationship as the fixed effect, familiarity and saturation as covariates, and image and participant as random effects. The odds of an item being rated into the medium over low bin were 2.17 times higher for home foods than for foreign foods, *z =* 14.69, *SE =* 0.05, *p < *.001, and the odds of it being assigned into the high over medium bin were 2.55 times higher for home foods than for foreign foods, *z =* 12.65, *SE =* 0.07, *p < *.001.

**Table 2:** Average % of images rated as low, medium, or high in nostalgic value by food relationship across all participants in the study. Home foods were more likely to be rated high in nostalgia than foreign foods.

Food relationship	Nostalgic value bin	% of Images rated (average)
**Home**	High	36.17
	Medium	27.48
	Low	36.35
**Foreign**	High	8.49
	Medium	16.63
	Low	76.27

### Validity of image set for nostalgic value

To validate the image set and the resulting nomothetic patterns, we established construct validity by looking at divergent and convergent validity.

### Divergent and convergent validity with familiarity and trait nostalgia

**Fig. 7:**
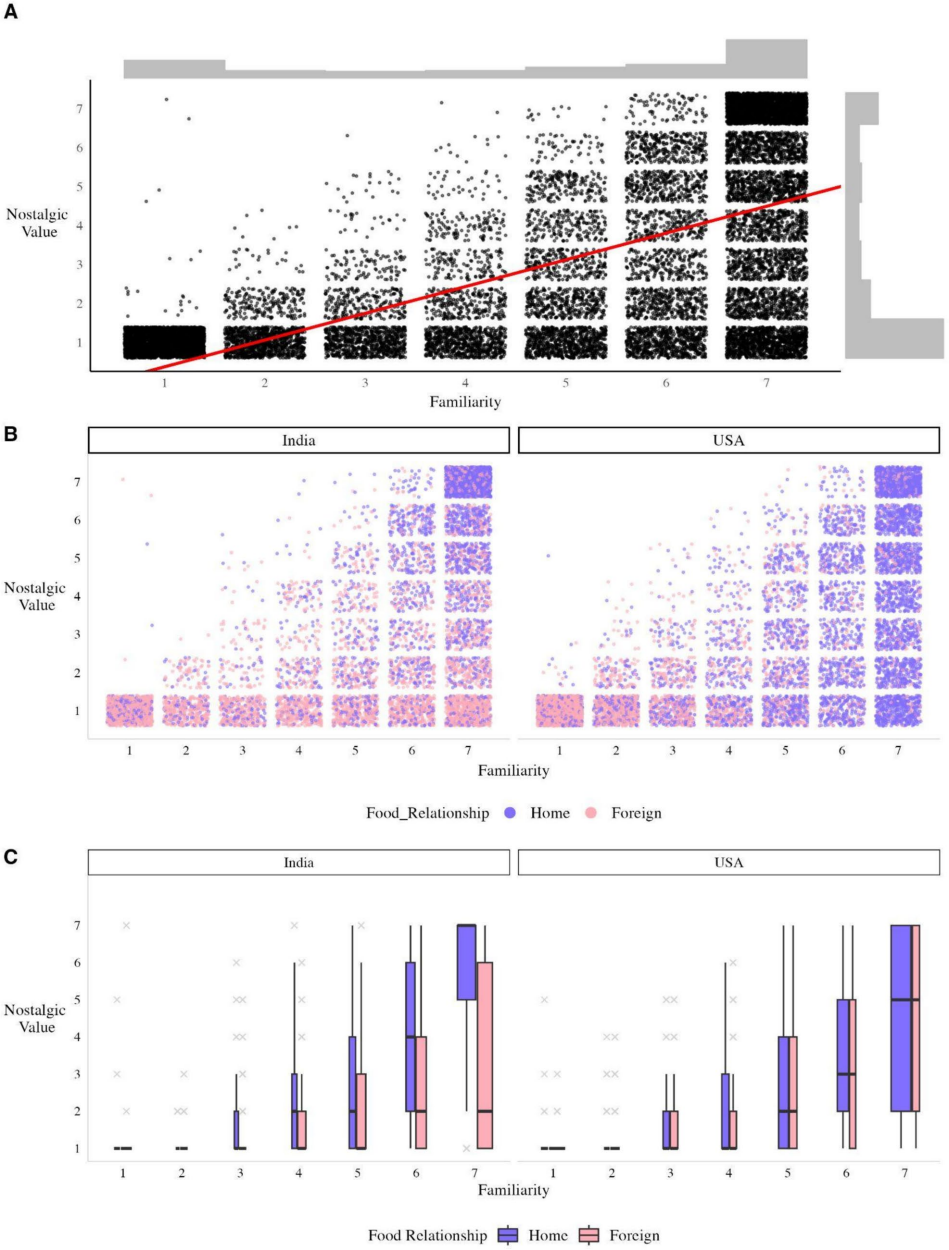
Relationship between food familiarity and nostalgic value, further separated by home country and food relationship*.* **A** Joint and marginal distribution of food familiarity and nostalgic value of all trials. The red line marks the slope and intercept from the model. As familiarity decreases, so does nostalgic value. Gray histograms are the marginal distributions for each variable. Both ratings were bimodally distributed toward the endpoints. **B** Joint distribution separated by home country (India on left, USA on right) and food relationship (home in purple, foreign in pink). **C** Boxplot with trials aggregated into median, range, and outliers (gray crosses). The width of the box marks the number of trials at each level of familiarity.

We had hypothesized that developmental context and specifically the larger displacement from home would result in greater nostalgic value ratings for Indians. To test whether familiarity was necessary but insufficient to evoke nostalgia, we looked at asymmetries in familiarity and nostalgic value between groups across items in a set of linear mixed-effects models, with images and participants as random effects. When using a model with only familiarity to predict nostalgic value, we found, as expected, that lower familiarity ratings coincided with lower nostalgic values, *t*(18,380)* =*117.7, *p < *.001, η_p_^2^ = .43, but high familiarity was not sufficient for evoking nostalgia (Fig. 7A).

To assess what predicts familiarity, we used home country and food relationship as fixed effects. Developmentally consistent foods, i.e., home foods, were largely more familiar, *F*(1, 207.11)* =* 100.24, *p < *.001, η_p_^2^ = .27. As further expected, given the current context (in the USA), there was also a significant interaction with home country: Indian participants were more familiar with foreign foods (i.e., US foods) than US Americans (i.e., Indian foods), *F*(1, 192.03)* =* 31.48*, p < *.001*,* η_p_^2^ = .14. We then estimated nostalgic value using home country and food relationship, with familiarity and saturation as nuisance covariates (Fig. 7B). Indians and US Americans found home foods to be higher in nostalgic value, *F*(1, 266.9)* =* 782.92, *p < .*001, η_p_^2^ = .07. The model also revealed a significant interaction, *F*(1, 197.6) = 189.50, *p* < .001, η_p_^2^ = .49. Indians reported significantly more nostalgic value for home foods than their US American peers, *b =* 1.04, *SE =* 0.16, *z =* 6.72, *p < *.001, but less nostalgic value for foreign foods than US Americans, *b* = −0.55, *SE =* 0.16, *z =* −3.56, *p < *.001. Despite higher familiarity with foreign foods than US Americans, Indians’ nostalgic value for these foods was lower than their US American counterparts (Fig. 7C).

We next examined the role of global trait nostalgia on our measurements of food-evoked nostalgia. We assessed group differences in trait nostalgia with a simple linear model estimating home country differences in global trait nostalgia. Indians and US Americans did not differ in their levels of trait nostalgia, *F*(1, 49) = 0.70, *p = .*41, η_p_^2^ = .01. Thus, trait nostalgia did not account for greater food-evoked nostalgia in Indian participants. Using linear mixed-effects models, we estimated participants’ state nostalgia proneness and state nostalgia magnitude for food images from global trait nostalgia, with food relationship as a covariate and participant as a random effect. Food relationship was necessary in the models, as state nostalgia proneness and state nostalgia magnitude scores were calculated separately for home and foreign foods. An individual’s global trait nostalgia had a medium-sized effect on state nostalgia magnitude, *t*(48)* =* 2.10*, p = *.04, η_p_^2^ = .08, and nostalgia proneness to food images, *t*(44.36)* =* 2.17, *p = .*04, η_p_^2^ = .10.

## Discussion

Experimental methods struggle in capturing phenomena shaped by developmental history and current context. In this study, we developed a method that balances experimental control with the contextual specificity required to capture meaningful individual variation in nostalgia as cued by foods. Capitalizing on the shared current context but differing developmental context of two cultural groups, we built an iterative qualitative and quantitative method which produced a large, standardized cross-cultural image set to evoke the emotion of nostalgia. We further researched the idiographic and nomothetic factors underlying its expression. Findings indicate that the picture stimuli were evocative. They effectively elicited hunger and held high nostalgic value. Nostalgic value exhibited an idiographically nomothetic pattern: vast variances between individuals on what evoked nostalgia, with a bias for developmentally consistent home foods, but high within-rater agreement over time. Our results highlight the need for a method that leans on context to simultaneously permit idiographic and nomothetic investigations. Our protocol offers a framework for creating stimuli tailored to specific contexts and emotions. Below, we discuss the contributions of the project to the study of the complex emotion of nostalgia and offer recommendations for the application of our stimuli set to psychophysiology methods requiring greater control and generalizability.

### Idiographic-nomothetic procedure creates culturally sensitive triggers for emotions

Our findings underscore the potential for manipulating a context-sensitive emotion in an empirical, ecological, and generalizable manner. Previous research either used completely homogeneous stimuli or self-selected stimuli, limiting ecological validity and generalizability, respectively (Maghbouleh, 2010; Merchant et al., 2013; Michels-Ratliff & Ennis, 2016; Paul, 2010; Petratou et al., 2020; Simpson et al., 2024). Our systematic operationalization of visual food stimuli with specific populations in mind (Fig. 1) helped to balance methodological standardization with ecological validity. Other food databases focus on culturally homogeneous images stripped of contextual details (Charbonnier et al., 2016; Gautier et al., 2019; Miccoli et al., 2014). Our standardization process retained natural backgrounds and objects (e.g., utensils on the dining table) in the curated images (Box 1) to reflect real-life food presentations and represent specific yet varying cultural foods. By choosing complex content for ecological relevance and controlling visual properties to reduce confounds, we created a standardized yet naturalistic image set. The resulting large stimuli set enabled sufficient trial numbers for statistical power—a common limitation of smaller standardized sets or self-selected stimuli (Wildschut & Sedikides, 2024). In addition, its multicultural food representation supports cross-cultural comparisons of nostalgia, addressing a key shortcoming of monocultural stimulus databases.

The cultural backgrounds of our participants helped disentangle a common conundrum in nostalgia research: differentiating familiarity and nostalgia. Familiarity predicts nostalgia across sensory domains—familiar songs, scents, and foods are more likely to evoke nostalgia (Mahon & Roth, 2023; Petratou et al., 2020; Reid et al., 2023). Because of this, at the measurement level, it can look as if familiarity is inseparable from nostalgia. However, we found that despite Indians’ greater familiarity with foreign (US American) foods compared to US Americans with foreign (Indian) foods, they did not rate these foods as more nostalgic. If familiarity were sufficient, Indians’ greater familiarity with foreign foods would translate into greater nostalgia for foreign foods. From a phenomenological perspective, there is a difference between nostalgia and familiarity, but with our use of two cultural groups, one of which was highly familiar with the foods of the other group, we were able to *measure* that difference.

### Contributions to the study of nostalgia

Aside from offering a method, this study offers multiple contributions to the literature on nostalgia. Our findings suggest that nostalgia evoked by food is highly idiographic and yet culturally specific and stable over time. Previous research has maintained that “universal” triggers could reliably evoke nostalgia in homogeneous groups (Oba et al., 2016), while others suggest that this is not possible, as nostalgia is a “transient mood state” (Batcho, 1995). We found some restricted evidence for universal triggers, with only six items consistently ranking as highly nostalgic among our participant groups (Fig. 6). Instead, participants on average found 21.02% of the images highly nostalgic, but which images were highly nostalgic was idiographic. Individuals had vastly different profiles of nostalgic magnitude for each image even within their cultural group (Fig. 4). This supports the highly idiographic view of nostalgia. However, this must be balanced with nomothetic findings as well: home foods were more nostalgic than foreign foods for both cultural groups across samples (Fig. 7). Rather than contradictions, these data align with research showing that nostalgia often anchors adults’ identity to their childhood (Debus, 2007). While the individual experience may be highly idiographic (e.g., mom’s cooking), it occurs within a cultural context that is shared by others, increasing the likelihood that individuals will share general cultural experiences (e.g., Indian cooking). We found that these idiographic-nomothetic findings were stable over time (Fig. 3). Our reliability is comparable to or exceeds the few other gold-standard emotion elicitation stimuli sets that measure test–retest reliability (Kim et al., 2018). Aside from supporting views of nostalgia as autobiographical (Green et al., 2023) and therefore stable, it also allows us to use pretesting before applying an idiographic-nomothetic approach to more expensive methods (see Applications section).

An important finding was the generally larger state nostalgia magnitude among Indian participants. We hypothesized that Indians currently in the USA would experience more nostalgia. One interpretation of this finding could be that Indians are simply culturally more nostalgic. However, there was no difference in our samples on trait nostalgia, corroborating findings from much larger studies using the same trait scale, which found no country-level differences (Hepper et al., 2024). Further, Indians were not more nostalgic for US foods, despite high familiarity with them. Therefore, greater displacement and especially lower access to these foods may be a better explanation for the nostalgic value of a food to an individual. For example, many of the US participants traveled from different hometowns, and therefore both cultural groups are technically displaced from their specific homes. However, Indians have much further to travel and are likely to experience more difficulty finding culturally important foods than their US American counterparts. This explains why Indian participants had higher nostalgic values. It also suggests that one underlying factor explaining even within-group differences in nostalgia could be related to the ease with which an individual can access the nostalgic item. Future research will need to disentangle this as it has implications for well-being. Previous research suggests that emotional attachment to familiar home objects, particularly in a foreign context, helps maintain identity and mitigate the psychological challenges of displacement (Kasstan, 2015; Wildschut et al., 2019). These nostalgic values may therefore signal some coping processes related to displacement from a homelike context.

Finally, we documented empirically that trait nostalgia has a relationship with state nostalgic ratings, but that this may interact with displacement from the developmental and cultural context. Our method produced a culturally sensitive set of food stimuli that received high nostalgic value ratings even from those with low trait nostalgia. To put it in mathematical terms, state nostalgia is not an injective function of trait nostalgia and may be more strongly associated with context and stimuli. It is possible that the antecedents of state and trait nostalgia are separable (Newman & Sachs, 2020; Ye et al., 2013), or a different trait nostalgia measure capturing everyday nostalgia ratings might show more convergence. Nostalgia may also differ according to the domain of inquiry or the sensory triggers used. Therefore, global trait measures of nostalgia may have much less correspondence with sensory-specific measures (e.g., smells, tastes, sounds) of nostalgic states. Future studies will need to explore these possibilities further.

### Applications: Using this method for idiographically nomothetic studies

We refer to our design approach as *idiographically nomothetic*: it begins with individual, context-rich experiences and organizes them into frameworks that support cross-participant comparison and statistical generalization. By translating idiographic responses into standardized paradigms, researchers can construct participant-sensitive designs that are both ecologically valid and suitable for group-level inference. While the broader method can be used “from scratch” to generate stimuli for other emotions, in this section, we will offer recommendations and applications related to our research question and stimuli set. All materials and rating tools are available online [https://github.com/hetvidoshi/contextsensitivestimulimethod.git], enabling replication or adaptation across contexts. We provide recommendations for participant and stimuli selection in the creation of a functional magnetic resonance imaging (fMRI) research study on nostalgia cued from food images. These images were curated for university students from the USA and India and may not be appropriate outside of these populations.

For participant selection, we recommend criterion-based purposive sampling using pretesting. Our study suggests that not all individuals reliably experience nostalgia in response to food stimuli at the same level, and trait-level nostalgia scores are only weakly predictive of image-based responses. However, because there is strong test–retest reliability, researchers can pretest large numbers of participants on these image sets and choose participants based on enough images reaching a high nostalgic rating. In fMRI design, at least 20 to 30 repetitions are recommended per condition in order to reliably model blood-oxygenation-level-dependent (BOLD) signals (Huettel et al., 2014). This does not mean that each participant must identify 20 unique nostalgic stimuli—stimuli can be repeated with careful consideration of habituation and order effects—but sufficient high-nostalgia and control trials are essential. Block designs, where images are clustered together in nostalgia-inducing versus non-nostalgia-inducing sequences, may tolerate fewer trials but require greater contrast between conditions. Event-related designs, where conditions are scrambled, will need a higher number of trials per participant, increasing the need for more nostalgic stimuli as cues. Based on our data, participants rated an average of 21% (40 of 194) images as highly nostalgic, and only 11% (11 of 98) of participants failed to rate at least 10 images as nostalgia-inducing. This suggests that for a target fMRI sample of 50 participants, researchers should anticipate pretesting at least approximately 15% more individuals to account for ineligibility due to insufficient nostalgic responsiveness.

For stimulus selection, researchers may adopt a fully idiographic design (e.g., personalized nostalgic and neutral images for each participant) or a pseudo-idiographic approach using a fixed set of standardized images with participant-specific categorization. Researchers have successfully completed idiographic designs, as is the case for research on romantic partners (Hsu et al., 2020), though this is rare and can introduce third variables. In contrast, a pseudo-idiographic approach is more controlled but involves using computational methods. For example, narrowing down the image set may be approached as a constraint satisfaction problem: finding a subset of participants whose individual ratings allow for the construction of personalized condition labels, using a shared set of images, while ensuring enough trials per condition for each person. In an AB design where condition A includes high-nostalgia, high-familiarity images and condition B includes low-nostalgia, high-familiarity images, each participant’s image ratings determine condition assignment. The same images are shown across individuals, but the categorization of those images depends on the individual. This preserves stimulus consistency while retaining subjective categorization, facilitating both within- and between-subject comparisons. Researchers can run permutation-based sampling or greedy search algorithms (LaFleur & Greevy, 2009; Wilt et al., 2010) to identify sets that maximize participant inclusion while meeting condition constraints. Larger pretest samples increase the feasibility of this method by enabling participant–image matching and cluster identification. However, in our test–retest sample, we had an attrition of 71.7% over 2 months. Pretest data would need to be collected in a short time, the algorithm run immediately, and the participants quickly contacted and screened for fMRI safety.

## Limitations and future directions

Various researchers have called for embracing context in psychological science (Cikara et al., 2022; Gonzalez & Rice, 2024) and heterogeneity in psychological science (Bryan et al., 2021) towards a different kind of generalizability (Thomas, 2023). Our method supports these calls by providing an idiographic-nomothetic approach to nostalgia cued by foods specifically, but also a general framework for understanding dynamic and situated processes like emotions (De Luca Picione, 2015). Nevertheless, there are several limitations that should be considered for future work. In terms of the resulting image set, our method holds strong ecological validity, but only insofar as it is used within the context it was created for. Food cultures evolve rapidly across time and space, and what elicits nostalgia for one population may hold no meaning—or even signal something entirely different—for another. Thus, the validity of our stimuli is ecological in the sense that it only arises when the relationship between the population and the stimuli is maintained. Like all organisms, human behavior is shaped by materiality, agency, and historicity (Gomez-Marin & Ghazanfar, 2019), and stimulus sets must be tailored accordingly. Our image set may work “out of the box,” but we believe it is best used to bootstrap further refinement sensitive to the specific research location. Second, the work could also include greater granularity on the US developmental context, which can include microclimates and cultures that are infused into the majority culture (e.g., Mexican food in southern California). Third, we need to validate our assumption that the participant's rating of nostalgic value reflects state nostalgia. This can be done by including other measures like ecological momentary assessments (Newman & Stone, 2019) and psychophysiology. Although we measured lower-level visual features of our food images, the latter types of investigations may be needed to remove spectral differences and other factors that may impact instrument specificity. Finally, in terms of accounting for possible mechanisms of nostalgia, future work should directly assess the current food/cultural context to assay what foods are available and not available to further test contextual hypotheses. These future directions would not only address questions on the nature of nostalgia and the potential special role of food in its evocation but also continue to develop approaches that balance phenomenological complexity with positivist generalizability.

## Supplementary Information

Below is the link to the electronic supplementary material.

Supplementary file1 (DOCX 311 KB)

Supplementary file2 (XLSX 52 KB)

Supplementary file3 (XLSX 21 KB)

## Data Availability

All data, stimuli, supplements, and other materials can be found at [https://github.com/hetvidoshi/contextsensitivestimulimethod.git.]

## Code availability

All scripts can be found at [https://github.com/hetvidoshi/contextsensitivestimulimethod.git.]

## References

[CR1] Abeyta, A. A., Corley, D., & Hasna, N. (2024). Nostalgia promotes positive beliefs about college belonging and success among first-generation college students. *International Journal of Applied Positive Psychology,**9*(2), 1–17. 10.1007/s41042-024-00163-4

[CR2] Appelbaum, M., Cooper, H., Kline, R. B., Mayo-Wilson, E., Nezu, A. M., & Rao, S. M. (2018). Journal article reporting standards for quantitative research in psychology: The APA Publications and Communications Board task force report. *American Psychologist,**73*(1), 3. 29345484 10.1037/amp0000191

[CR3] Asghar, M. (2018). Familial nostalgia. *European Journal of Media Art and Photography,**6*(1), 92–98.

[CR4] Avery, J. A., Liu, A. G., Ingeholm, J. E., Gotts, S. J., & Martin, A. (2021). Viewing images of foods evokes taste quality-specific activity in gustatory insular cortex. *Proceedings of the National Academy of Sciences*. 10.1073/pnas.2010932118 10.1073/pnas.2010932118 PMC7812770 33384331

[CR5] Barlow, D. H., & Nock, M. K. (2009). Why can’t we be more idiographic in our research? *Perspectives on Psychological Science,**4*(1), 19–21. 10.1111/j.1745-6924.2009.01088.x 26158824 10.1111/j.1745-6924.2009.01088.x

[CR6] Barrett, F. S., Grimm, K. J., Robins, R. W., Wildschut, T., Sedikides, C., & Janata, P. (2010). Music-evoked nostalgia: Affect, memory, and personality. *Emotion,**10*, 390–403. 10.1037/a0019006 20515227 10.1037/a0019006

[CR7] Barrett, L. F. (2006). Are emotions natural kinds? *Perspectives on Psychological Science,**1*(1), 28–58. 10.1111/j.1745-6916.2006.00003.x 26151184 10.1111/j.1745-6916.2006.00003.x

[CR8] Batcho, K. I. (1995). Nostalgia: A psychological perspective. *Perceptual and Motor Skills,**80*(1), 131–143. 10.2466/pms.1995.80.1.131 7624184 10.2466/pms.1995.80.1.131

[CR9] Baxter, J. E. (2016). Adult nostalgia and children’s toys past and present. *International Journal of Play,**5*(3), 230–243. 10.1080/21594937.2016.1220046

[CR10] Beltz, A. M., Wright, A. G. C., Sprague, B. N., & Molenaar, P. C. M. (2016). Bridging the nomothetic and idiographic approaches to the analysis of clinical data. *Assessment,**23*(4), 447–458. 10.1177/1073191116648209 27165092 10.1177/1073191116648209 PMC5104664

[CR11] Blechert, J., Meule, A., Busch, N. A., & Ohla, K. (2014). Food-pics: an image database for experimental research on eating and appetite [Original Research]. *Frontiers in Psychology*,* 5*. 10.3389/fpsyg.2014.00617 10.3389/fpsyg.2014.00617 PMC4067906 25009514

[CR12] Bonikowski, B., & Stuhler, O. (2022). Reclaiming the Past to Transcend the Present: Nostalgic Appeals in U.S. Presidential Elections. *Sociological Forum,**37*(S1), 1263–1293. 10.1111/socf.12838

[CR13] Bryan, C. J., Tipton, E., & Yeager, D. S. (2021). Behavioural science is unlikely to change the world without a heterogeneity revolution. *Nature Human Behaviour,**5*(8), 980–989. 10.1038/s41562-021-01143-3 34294901 10.1038/s41562-021-01143-3 PMC8928154

[CR14] Charbonnier, L., van Meer, F., van der Laan, L. N., Viergever, M. A., & Smeets, P. A. M. (2016). Standardized food images: A photographing protocol and image database. *Appetite,**96*, 166–173. 26344127 10.1016/j.appet.2015.08.041

[CR15] Cho, H. (2023). Nostalgia in sport and leisure. *Current Opinion in Psychology,**49*, 101551. 10.1016/j.copsyc.2022.101551 36702009 10.1016/j.copsyc.2022.101551 PMC9870724

[CR16] Chrostowska, S. D. (2010). Consumed by Nostalgia? *SubStance,**39*(2), 52–70. Retrieved July 1, 2024 from https://www.jstor.org/stable/40801075

[CR17] Cikara, M., Martinez, J. E., & Lewis, N. A. (2022). Moving beyond social categories by incorporating context in social psychological theory. *Nature Reviews Psychology,**1*(9), 537–549. 10.1038/s44159-022-00079-3

[CR18] Colla, K., Keast, R., Hartley, I., & Liem, D. G. (2021). Using an online photo based questionnaire to predict tasted liking and amount sampled of familiar and unfamiliar foods by female nutrition students. *Journal of Sensory Studies,**36*(1), e12614. 10.1111/joss.12614

[CR19] De Luca Picione, R. (2015). The Idiographic approach in psychological research. The challenge of overcoming old distinctions without risking to homogenize. *Integrative Psychological & Behavioral Science,**49*(3), 360–370. 10.1007/s12124-015-9307-5 25939530 10.1007/s12124-015-9307-5

[CR20] Debus, D. (2007). Being emotional about the past: On the nature and role of past-directed emotions*. *Noûs,**41*(4), 758–779. 10.1111/j.1468-0068.2007.00669.x

[CR21] Duruz, J. (1999). Food as nostalgia: Eating the Fifties and Sixties. *Australian Historical Studies,**30*(113), 231–250. 19391302 10.1080/10314619908596100

[CR22] Elgenius, G., & Rydgren, J. (2022). Nationalism and the Politics of Nostalgia. *Sociological Forum,**37*(S1), 1230–1243. 10.1111/socf.12836

[CR23] Fan, Y., Jiang, J., & Hu, Z. (2020). Abandoning distinctiveness: The influence of nostalgia on consumer choice. *Psychology & Marketing,**37*(10), 1342–1351. 10.1002/mar.21370

[CR24] Ferreira, T., & Rasband, W. (2011). *ImageJ user guide*. National Institutes of Health.

[CR25] Foroni, F., Pergola, G., Argiris, G., & Rumiati, R. (2013). The FoodCast research image database (FRIDa) [Original Research]. *Frontiers in Human Neuroscience*,* 7*. 10.3389/fnhum.2013.00051 10.3389/fnhum.2013.00051 PMC3585434 23459781

[CR26] Gates, K. M., & Molenaar, P. C. (2012). Group search algorithm recovers effective connectivity maps for individuals in homogeneous and heterogeneous samples. *NeuroImage,**63*(1), 310–319. 10.1016/j.neuroimage.2012.06.026 22732562 10.1016/j.neuroimage.2012.06.026

[CR27] Gautier, Y., Meurice, P., Coquery, N., Constant, A., Bannier, E., Serrand, Y., Ferré, J.-C., Moirand, R., & Val-Laillet, D. (2019). Implementation of a new food picture database in the context of fmri and visual cognitive food-choice task in healthy volunteers. *Frontiers in Psychology*. 10.3389/fpsyg.2019.02620 31849751 10.3389/fpsyg.2019.02620 PMC6902029

[CR28] Geniusas, S. (2025). Nostalgia for the past, present and future. *Emotion Review,* , Article 17540739251323528. 10.1177/17540739251323528

[CR29] Gest, J., Reny, T., & Mayer, J. (2017). Roots of the radical right: Nostalgic deprivation in the United States and Britain. *Comparative Political Studies,**51*(13), 1694–1719. 10.1177/0010414017720705

[CR30] Gomez-Marin, A., & Ghazanfar, A. A. (2019). The life of behavior. *Neuron,**104*(1), 25–36. 10.1016/j.neuron.2019.09.017 31600513 10.1016/j.neuron.2019.09.017 PMC6873815

[CR31] Gonzalez, M. Z., & Rice, M. A. (2024). Behavioural sciences need behavioural ecology. *Nature Human Behaviour,**8*(7), 1240–1242. 10.1038/s41562-024-01906-8 38802542 10.1038/s41562-024-01906-8

[CR32] Green, J. D., Reid, C. A., Kneuer, M. A., & Hedgebeth, M. V. (2023). The proust effect: Scents, food, and nostalgia. *Current Opinion in Psychology,**50*, Article 101562. 10.1016/j.copsyc.2023.101562 36863096 10.1016/j.copsyc.2023.101562

[CR33] Gross, J. J. (2015). Emotion regulation: Current status and future prospects. *Psychological Inquiry,**26*(1), 1–26. 10.1080/1047840X.2014.940781

[CR34] Hepper, E. G., & Dennis, A. (2023). From rosy past to happy and flourishing present: Nostalgia as a resource for hedonic and eudaimonic wellbeing. *Current Opinion in Psychology,**49*, Article 101547. 10.1016/j.copsyc.2022.101547 36640677 10.1016/j.copsyc.2022.101547

[CR35] Hepper, E. G., Ritchie, T. D., Sedikides, C., & Wildschut, T. (2012). Odyssey’s end: Lay conceptions of nostalgia reflect its original homeric meaning. *Emotion,**12*, 102–119. 10.1037/a0025167 21859192 10.1037/a0025167

[CR36] Hepper, E. G., Sedikides, C., Wildschut, T., Cheung, W. Y., Abakoumkin, G., Arikan, G., Aveyard, M., Baldursson, E. B., Bialobrzeska, O., Bouamama, S., Bouzaouech, I., Brambilla, M., Burger, A. M., Chen, S. X., Cisek, S., Demassosso, D., Estevan-Reina, L., González Gutiérrez, R., Gu, L., … Zengel, B. (2024). Pancultural nostalgia in action: Prevalence, triggers, and psychological functions of nostalgia across cultures. *Journal of Experimental Psychology: General,**153*(3), 754–778. 10.1037/xge0001521 38252088 10.1037/xge0001521

[CR37] Hillman, J. (1975). Pothos: The nostalgia of the puer eternus. *Loose Ends: Primary papers in archetypal psychology* (pp. 49–62). Spring Publications.

[CR38] Hofer, J. (1688). *Disertatio medica de nostalgia*. Typis Iacobi Bertschii.

[CR39] Hollis, J. H. (2022). The Use of Questionnaires to Measure Appetite. In C. B. Betim Cazarin (Ed.), *Basic Protocols in Foods and Nutrition* (pp. 249–263). Springer US. 10.1007/978-1-0716-2345-9_16

[CR40] Hsu, D. T., Sankar, A., Malik, M. A., Langenecker, S. A., Mickey, B. J., & Love, T. M. (2020). Common neural responses to romantic rejection and acceptance in healthy adults. *Social Neuroscience,**15*(5), 571–583. 10.1080/17470919.2020.1801502 32715953 10.1080/17470919.2020.1801502 PMC7674199

[CR41] Huettel, S. A., Song, A. W., & McCarthy, G. (2014). *Functional Magnetic Resonance Imaging* (3rd ed.). Sinauer. Retrieved November 15, 2025 from https://books.google.com/books?id=CUrVoAEACAAJ

[CR42] Hurtado-Parrado, C., & López-López, W. (2015). Single-case research methods: History and suitability for a psychological science in need of alternatives. *Integrative Psychological & Behavioral Science,**49*(3), 323–349. 10.1007/s12124-014-9290-2 25876996 10.1007/s12124-014-9290-2

[CR43] Ikeda, H., & Kusumi, T. (2023). Episodic memory and personal semantics as triggers of nostalgia: Its relationships between abstraction of memory content and temporal distance. *Memory,* , Article 2196038. 10.1080/09658211.2023.2196038 10.1080/09658211.2023.2196038 37000614

[CR44] Ismail, S. U., Cheston, R., Christopher, G., & Meyrick, J. (2020). Nostalgia as a psychological resource for people with dementia: A systematic review and meta-analysis of evidence of effectiveness from experimental studies. *Dementia (London),**19*(2), 330–351. 10.1177/1471301218774909 29747526 10.1177/1471301218774909

[CR45] Jacobsen, M. H. (2023). The sociology of nostalgia. *Current Opinion in Psychology,**50*, 101556. 10.1016/j.copsyc.2023.101556 36774853 10.1016/j.copsyc.2023.101556

[CR46] Janata, P., Tomic, S. T., & Rakowski, S. K. (2007). Characterisation of music-evoked autobiographical memories. *Memory,**15*(8), 845–860. 10.1080/09658210701734593 17965981 10.1080/09658210701734593

[CR47] Kasstan, B. (2015). The taste of trauma reflections of ageing Shoah survivors on food and how they (re) inscribe it with meaning. *Scripta Instituti Donneriani Aboensis,**26*, 349–365. 10.30674/scripta.67461

[CR48] Kersten, M., Swets, J. A., Cox, C. R., Kusumi, T., Nishihata, K., & Watanabe, T. (2020). Attenuating Pain With the Past: Nostalgia Reduces Physical Pain [Brief Research Report]. *Frontiers in Psychology*, *11*. 10.3389/fpsyg.2020.572881 10.3389/fpsyg.2020.572881 PMC7589743 33154729

[CR49] Kim, H., Lu, X., Costa, M., Kandemir, B., Adams, R. B., Li, J., Wang, J. Z., & Newman, M. G. (2018). Development and validation of image stimuli for emotion elicitation (ISEE): A novel affective pictorial system with test-retest repeatability. *Psychiatry Research,**261*, 414–420. 10.1016/j.psychres.2017.12.068 29353766 10.1016/j.psychres.2017.12.068 PMC6510029

[CR50] Klemm, A., & Miura, K. (2022). Batch processing methods in imageJ. *Bioimage data analysis workflows-advanced components and methods* (pp. 7–27). Springer International Publishing Cham.

[CR51] Knebel, J.-F., Toepel, U., Hudry, J., le Coutre, J., & Murray, M. M. (2008). Generating controlled image sets in cognitive neuroscience research. *Brain Topography,**20*(4), 284–289. 10.1007/s10548-008-0046-5 18338244 10.1007/s10548-008-0046-5

[CR52] Kreibig, S. D. (2010). Autonomic nervous system activity in emotion: A review. *Biological Psychology,**84*(3), 394–421. 10.1016/j.biopsycho.2010.03.010 20371374 10.1016/j.biopsycho.2010.03.010

[CR53] Krippendorff, K. (2011). *Computing Krippendorff’s alpha-reliability*. Annenberg School for Communication Departmental Papers.

[CR54] LaFleur, B. J., & Greevy, R. A. (2009). Introduction to permutation and resampling-based hypothesis tests∗. *Journal of Clinical Child & Adolescent Psychology,**38*(2), 286–294. 10.1080/15374410902740411 19283606 10.1080/15374410902740411

[CR55] Lamiell, J. T. (1998). `Nomothetic’ and `Idiographic’: Contrasting Windelband’s understanding with contemporary usage. *Theory & Psychology,**8*(1), 23–38. 10.1177/0959354398081002

[CR56] Layous, K., Kurtz, J. L., Wildschut, T., & Sedikides, C. (2022). The effect of a multi-week nostalgia intervention on well-being: Mechanisms and moderation. *Emotion,**22*(8), 1952–1968. 10.1037/emo0000817 34591502 10.1037/emo0000817

[CR57] Lee, A., & Hood, B. (2021). The origins and development of attachment object behaviour. *Current Opinion in Psychology,**39*, 72–75. 10.1016/j.copsyc.2020.07.023 32841814 10.1016/j.copsyc.2020.07.023

[CR58] Lerner, J. S., Li, Y., Valdesolo, P., & Kassam, K. S. (2015). Emotion and decision making. *Annual Review of Psychology,**66*(1), 799–823. 10.1146/annurev-psych-010213-115043 25251484 10.1146/annurev-psych-010213-115043

[CR59] Li, X. P., Kong, W. H., & Yang, F. X. (2021). Authentic food experiences bring us back to the past: An investigation of a local food night market. *Journal of Travel & Tourism Marketing,**38*(3), 233–246. 10.1080/10548408.2021.1902910

[CR60] Lim, A. J., Teo, P. S., Tan, V. W., & Forde, C. G. (2020). Associations between psycho-hedonic responses to sweet and savoury tastes with diet and body composition in a sample of Asian females. *Foods,**9*(9), Article 1318. 32962029 10.3390/foods9091318 PMC7555575

[CR61] Maghbouleh, N. (2010). “Inherited nostalgia” among second-generation Iranian Americans: A case study at a Southern California University [Article]. *Journal of Intercultural Studies,**31*(2), 199–218. 10.1080/07256861003606382

[CR62] Mahon, A. J. S., & Roth, E. A. (2023). What elicits music-evoked nostalgia? An exploratory study among college students. *Psychology of Music,**51*(1), 159–171. 10.1177/03057356221087446

[CR63] Mandal, S., Gunasekar, S., Dixit, S. K., & Das, P. (2022). Gastro-nostalgia: Towards a higher order measurement scale based on two gastro festivals. *Tourism Recreation Research,**47*(3), 293–315. 10.1080/02508281.2021.1951589

[CR64] Merchant, A., Latour, K., Ford, J. B., & Latour, M. S. (2013). How strong is the pull of the past? Measuring personal nostalgia evoked by advertising. *Journal of Advertising Research,**53*(2), 150–165. 10.2501/JAR-53-2-150-165

[CR65] Miccoli, L., Delgado, R., Rodríguez-Ruiz, S., Guerra, P., García-Mármol, E., & Fernández-Santaella, M. C. (2014). Meet OLAF, a good friend of the IAPS! The Open Library of Affective Foods: A tool to investigate the emotional impact of food in adolescents. *PLoS One,**9*(12), Article e114515. 10.1371/journal.pone.0114515 25490404 10.1371/journal.pone.0114515 PMC4260831

[CR66] Michels-Ratliff, E., & Ennis, M. (2016). This is your song: Using participants’ music selections to evoke nostalgia and autobiographical memories efficiently. *Psychomusicology: Music, Mind, and Brain,**26*, 379–384. 10.1037/pmu0000167

[CR67] Narchi, I., Walrand, S., Boirie, Y., & Rousset, S. (2008). Emotions generated by food in elderly French people. *Journal of Nutrition, Health & Aging,**12*(9), 626–633. 10.1007/BF03008273 PMC12892633 18953460

[CR68] Newman, D. B., & Sachs, M. E. (2020). The negative interactive effects of nostalgia and loneliness on affect in daily life. *Frontiers in Psychology,**11*, Article 2185. 10.3389/fpsyg.2020.02185 32982886 10.3389/fpsyg.2020.02185 PMC7492671

[CR69] Newman, D. B., & Stone, A. A. (2019). *Understanding daily life with Ecological Momentary Assessment* . Routledge/Taylor & Francis Group. 10.4324/9781351137713-14

[CR70] Niu, Y., Todd, R. M., Kyan, M., & Anderson, A. K. (2012). Visual and emotional salience influence eye movements. *ACM Transactions on Applied Perception,**9*(3), Article 13. 10.1145/2325722.2325726

[CR71] Oba, K., Noriuchi, M., Atomi, T., Moriguchi, Y., & Kikuchi, Y. (2016). Memory and reward systems coproduce ‘nostalgic’ experiences in the brain. *Social Cognitive and Affective Neuroscience,**11*(7), 1069–1077. 10.1093/scan/nsv073 26060325 10.1093/scan/nsv073 PMC4927028

[CR72] Paul, B. (2010). Nostalgia-inducing music and perceptions of social support satisfaction 11th international conference on music perception and cognition, Retrieved March 25, 2025 from https://www.researchgate.net/publication/235792445_Nostalgia-Inducing_Music_and_Perceptions_of_Social_Support_Satisfaction/citation/download

[CR73] Peirce, J. W. (2007). PsychoPy—psychophysics software in Python. *Journal of Neuroscience Methods,**162*(1–2), 8–13. 17254636 10.1016/j.jneumeth.2006.11.017 PMC2018741

[CR74] Pérez, J. M. M., & Pascau, J. (2013). *Image processing with ImageJ*. Packt Publishing Ltd.

[CR75] Petratou, E., Paradisi, N., Diamantis, O., & Stalikas, A. (2020). Psychological implications of nostalgic scents of childhood. *Psychology,**11*(12), 2066.

[CR76] Pinheiro J, Bates D, R Core Team (2025). nlme: Linear and Nonlinear Mixed Effects Models. *R package version, 3*, 1–168. 10.32614/CRAN.package.nlme; https://CRAN.R-project.org/package=nlme

[CR77] R Core Team (2024). R: A language and environment for statistical computing_. R Foundation for Statistical. *Computing*, Vienna. https://www.R-project.org/

[CR78] Reid, C. A., Green, J. D., Buchmaier, S., McSween, D. K., Wildschut, T., & Sedikides, C. (2023). Food-evoked nostalgia. *Cognition & Emotion,**37*(1), 34–48. 10.1080/02699931.2022.2142525 36331076 10.1080/02699931.2022.2142525

[CR79] Reid, C. A., Green, J. D., Wildschut, T., & Sedikides, C. (2015). Scent-evoked nostalgia. *Memory,**23*(2), 157–166. 10.1080/09658211.2013.876048 24456210 10.1080/09658211.2013.876048

[CR80] Richardson, J. T. E. (2011). Eta squared and partial eta squared as measures of effect size in educational research. *Educational Research Review,**6*(2), 135–147. 10.1016/j.edurev.2010.12.001

[CR81] Routledge, C., Arndt, J., Sedikides, C., & Wildschut, T. (2008). A blast from the past: The terror management function of nostalgia [Article]. *Journal of Experimental Social Psychology,**44*(1), 132–140. 10.1016/j.jesp.2006.11.001

[CR82] Rubin, D. C., & Schulkind, M. D. (1997). Distribution of important and word-cued autobiographical memories in 20-, 35-, and 70-year-old adults. *Psychology and Aging,**12*(3), 524–535. 10.1037/0882-7974.12.3.524 9308099 10.1037//0882-7974.12.3.524

[CR83] Salmose, N. (2019). Nostalgia makes us all tick: A special issue on contemporary nostalgia. *Humanities,**8*(3), 144.

[CR84] Scherer, K. R. (2005). What are emotions? And how can they be measured? *Social Science Information,**44*(4), 695–729. 10.1177/0539018405058216

[CR85] Sedikides, C., & Wildschut, T. (2018). Finding meaning in nostalgia. *Review of General Psychology,**22*(1), 48–61. 10.1037/gpr0000109

[CR86] Sedikides, C., & Wildschut, T. (2024). On the nature of nostalgia: A psychological perspective. *Emotion Review*. 10.1177/17540739241303497

[CR87] Sedikides, C., Wildschut, T., Arndt, J., & Routledge, C. (2008). Nostalgia: Past, present, and future. *Current Directions in Psychological Science,**17*(5), 304–307. 10.1111/j.1467-8721.2008.00595.x

[CR88] Sedikides, C., Wildschut, T., Routledge, Arndt, J., Hepper, E. G., & Zhou, X. Y. (2015). To Nostalgize: Mixing memory with affect and desire. In J. M. Olson & M. P. Zanna (Eds.), *Advances in Experimental Social Psychology* (vol. 51, pp. 189–273). Elsevier Academic Press Inc. 10.1016/bs.aesp.2014.10.001

[CR89] Sedikides, C., Wildschut, T., Routledge, C., & Arndt, J. (2015). Nostalgia counteracts self-discontinuity and restores self-continuity. *European Journal of Social Psychology,**45*(1), 52–61. 10.1002/ejsp.2073

[CR90] Simpson, K., Angus, D. J., & Lee, M. F. (2024). Nostalgic food heals for us’: A qualitative exploration of experiences with nostalgia, food, and mood. *Health Promotion Journal of Australia*. 10.1002/hpja.873 38772549 10.1002/hpja.873

[CR91] Smeekes, A., Wildschut, T., & Sedikides, C. (2021). Longing for the “good old days” of our country: National nostalgia as a new master-frame of populist radical right parties. *Journal of Theoretical Social Psychology,**5*(2), 90–102. 10.1002/jts5.78

[CR92] Smith, C. A., & Kirby, L. D. (2012). Affect and cognitive appraisal processes. *Handbook of affect and social cognition* (pp. 76–93). Psychology Press.

[CR93] Stice, E., Telch, C. F., & Rizvi, S. L. (2000). Development and validation of the eating disorder diagnostic scale: A brief self-report measure of anorexia, bulimia, and binge-eating disorder. *Psychological Assessment,**12*(2), 123–131. 10.1037/1040-3590.12.2.123 10887758 10.1037//1040-3590.12.2.123

[CR94] Sutton, D., & Vournelis, L. (2009). Vefa or Mamalakis: Cooking up nostalgia in contemporary Greece. *South European Society and Politics,**14*(2), 147–166. 10.1080/13608740903037851

[CR95] Tan, A., Rice, M. A., & Gonzalez, M. Z. (2024). A pivotal time and place: University place attachment, childhood neighborhood affordances, and internalizing symptoms in emerging adulthood. *Emerging Adulthood,**12*(4), 509–523. 10.1177/21676968241240186

[CR96] Thomas, A. K. (2023). Studying cognition in context to identify universal principles. *Nature Reviews Psychology,**2*(8), 453–454. 10.1038/s44159-023-00209-5

[CR97] Turner, R., Wildschut, T., & Sedikides, C. (2022). Reducing social distance caused by weight stigma: Nostalgia changes behavior toward overweight individuals. *Journal of Applied Social Psychology,**52*(6), 429–438. 10.1111/jasp.12869

[CR98] Vasvári, L. O. (2021). Culinary nostalgia and post-traumatic stress disorder: Addenda to Kinga Király’s az újrakezdés receptjei (2019) / Recipes for a New Beginning (2020). *Hungarian Cultural Studies,**14*, 186–204.

[CR99] Vázquez-Medina, J. A., & Medina, F. X. (2015). Migration, nostalgia and the building of a food imaginary: Mexican migrants at “La Pulga” Market in San Joaquin Valley, California. *ESSACHESS- Journal for Communication Studies,**2*, 133–146.

[CR100] Viladrich, A., & Tagliaferro, B. (2016). Picking fruit from our backyard’s trees: The meaning of nostalgia in shaping Latinas’ eating practices in the United States. *Appetite,**97*, 101–110. 10.1016/j.appet.2015.11.017 26593102 10.1016/j.appet.2015.11.017

[CR101] Wang, J. (2023). Nostalgia in tourism. *Current Opinion in Psychology,**49*, 101552. 10.1016/j.copsyc.2022.101552 36669250 10.1016/j.copsyc.2022.101552

[CR102] Wickham, H. (2016). *ggplot2: Elegant graphics for data analysis*. Springer-Verlag New York. Retrieved November 15, 2025 from https://ggplot2.tidyverse.org

[CR103] Wildschut, T., & Sedikides, C. (2023). Water from the Lake of Memory: The regulatory model of nostalgia. *Current Directions in Psychological Science,**32*(1), 57–64. 10.1177/09637214221121768

[CR104] Wildschut, T., & Sedikides, C. (2024). Psychology and nostalgia: A primer on experimental nostalgia inductions. *The Routledge handbook of nostalgia* (pp. 54–69). Routledge.

[CR105] Wildschut, T., Sedikides, C., & Alowidy, D. (2019). Hanin: Nostalgia among Syrian refugees [Article]. *European Journal of Social Psychology,**49*(7), 1368–1384. 10.1002/ejsp.2590

[CR106] Willenbockel, V., Sadr, J., Fiset, D., Horne, G. O., Gosselin, F., & Tanaka, J. W. (2010). Controlling low-level image properties: The SHINE toolbox. *Behavior Research Methods,**42*(3), 671–684. 10.3758/BRM.42.3.671 20805589 10.3758/BRM.42.3.671

[CR107] Wilt, C., Thayer, J., & Ruml, W. (2010). A comparison of greedy search algorithms. *Proceedings of the International Symposium on Combinatorial Search,**1*(1), 129–136. 10.1609/socs.v1i1.18182

[CR108] Wright, A. G. C., Gates, K. M., Arizmendi, C., Lane, S. T., Woods, W. C., & Edershile, E. A. (2019). Focusing personality assessment on the person: Modeling general, shared, and person specific processes in personality and psychopathology. *Psychological Assessment,**31*(4), 502–515. 10.1037/pas0000617 30920277 10.1037/pas0000617 PMC9380034

[CR109] Wulf, T., Bowman, N. D., Velez, J. A., & Breuer, J. (2020). Once upon a game: Exploring video game nostalgia and its impact on well-being. *Psychology of Popular Media,**9*(1), 83–95. 10.1037/ppm0000208

[CR110] Ye, S. Q., Ngan, R. Y. L., & Hui, A. N. N. (2013). The state, not the trait, of nostalgia increases creativity. *Creativity Research Journal,**25*(3), 317–323. 10.1080/10400419.2013.813797

[CR111] Zhou, X., van Tilburg, W. A. P., Mei, D., Wildschut, T., & Sedikides, C. (2019). Hungering for the past: Nostalgic food labels increase purchase intentions and actual consumption. *Appetite,**14.* 31077773 10.1016/j.appet.2019.05.007

