# Supplementary material for: Same emotion, different stimuli: A context-sensitive method to evoke nostalgia
Source: Behav Res Methods. 2026 Apr 7;58(4):105. doi: 10.3758/s13428-026-02971-9 (PMC13065580; doi:10.3758/s13428-026-02971-9)
Supplement: Supplementary file 1 — Supplementary file1 (DOCX 311 KB) [file 13428_2026_2971_MOESM1_ESM.docx]

**Appendices**

**Appendix A: Power Analysis**

*A priori effect and sample size.* We used the G*Power software ([Faul et al., 2009](#_ENREF_24)) to calculate implied power for an F test with repeated measures and within-between interaction, given a typical sample size, an *a priori* effect size, and a β/α ratio. Assuming a sample size of 20 participants from each country, an effect size of η_p_^2^ = .04 (selected from the smallest effect sizes found in recent nostalgia studies, e.g. Sidhu et al., 2025), 2 countries (India & USA), 83 measurements per person (the minimum images in each food category), and β/α ratio = 4, we find the power to be 1.00, which is greater than the threshold of 0.80. All our cohorts, of equal or greater sizes, are sufficiently powered to find any effect size of η_p_^2^ >= .04.

*Sensitivity.* We calculated the smallest effect size we could detect given a typical sample size, α = 0.05, and Power = 0.8. For a sample of 40 people, G*Power outputs the smallest effect size at f = 0.075/η_p_^2^ = .006. All our cohorts, of equal or greater sizes, are sufficiently powered to find effects of η_p_^2^ >= .006.

**Appendix B: Cohort-wise Breakdown**

**Cohort 1 Timepoint 1**

Forty-six healthy participants were recruited from the undergraduate and graduate student population at Cornell University. Participants were pre-screened via an online survey to ensure only those who grew up in India or the United States and presently live in the USA took part in the study. Twenty-one students from India (12 males) between the ages of 22 and 34 (*M* = 25.48, *SD* = 2.93) and twenty-five from the US (5 males) between the ages of 18 and 24 (*M = 20.*56, *SD =* 1.56) took part in the study. Race breakdown in the US American group was 12% African American, 4% Latino, 36% Asian (non-Indians), 40% Caucasian, and 8% Mixed. Participants received twelve dollars or two SONA credits. Data collection took place between February and June 2022.

**Cohort 1 Timepoint 2**

Of the 46, 33 subjects (India: 16 (6 females, 10 males); USA: 17 (14 females, 3 males)) returned at least 60 days (*M =* 113.48*, SD =* 32.04*)* after participation to repeat the study. Data from only the participants who returned were retained from S1 to measure test-retest reliability. Participants received identical compensation to S1. Data collection took place from July to October 2022.

**Cohort 2**

Fifty-two healthy participants were recruited identically. The pool consisted of 26 participants from the USA (2 males) aged 18 to 30 (*M =* 20.27*, SD =* 2.32*)* and 26 from India (12 males) between the ages of 22 and 62 (*M =* 28.88*, SD =* 10.73*)*. Race breakdown in the US American group was 8% African American, 31% Asian (non-Indians), 8% Hispanic, 38% Caucasian, and 15% Mixed. These subjects received twenty dollars or four SONA credits for participation because their study was an extended, modified version of study one (more details under evaluative procedures). Data collection took place between October 2022 and November 2023.

**Appendix C: Estimating Calories of Foods**

A calorie breakdown for each food item was collected (Supplement 3). The breakdown included carbohydrates, calories/energy, fats, sugar, proteins, and dietary fiber. It was easier to find standard, validated calories for USA than for India foods. Most of the information for the former category was sourced using the Food and Nutrient Database for Dietary Studies (FNDDS). To combat a lack of information on many local Indian foods, we collected their nutritional information from at least three online sources and averaged the values for each nutrient group. We are currently working on trying to acquire nutrient values for all Indian foods from the same source for better standardization using Azumio’s Calorie Mama API.

Images were then classified into low, medium, and high-calorie foods. A literature review ([Services, 2020](#_ENREF_90)) revealed that 40 calories are considered low, 100 is moderate, and 400 per serving is high. Three bins of 0-200, 200-400, and 400+ were used to classify the items accordingly. The rationale was to create a set of food items with a wide range of caloric values that are representative of an individual’s ‘food universe’.

**Appendix D: Exclusion of Images**

29 of 229 were alternate images, which were discarded *post hoc* because of their poor performance (relative to the original). Cohort 2 viewed the remaining 200 images, and an additional 17 new foods (totaling 102 India & 115 USA foods). This is our final set. Of the 200 images shared between the two cohorts, 6 Indian foods were removed since they were added halfway through the data collection. Only data from the 194 images (83 India Foods, 111 USA Foods) viewed by all participants is reported in this paper. We incidentally had more USA foods, so we factor in unequal variance in our analyses. (The final stimuli set that was used for Cohort 2 and subsequent studies can be found on the linked GitHub repository: <https://github.com/hetvidoshi/contextsensitivestimulimethod.git>).

**Appendix E: Adjusted ICC for models**


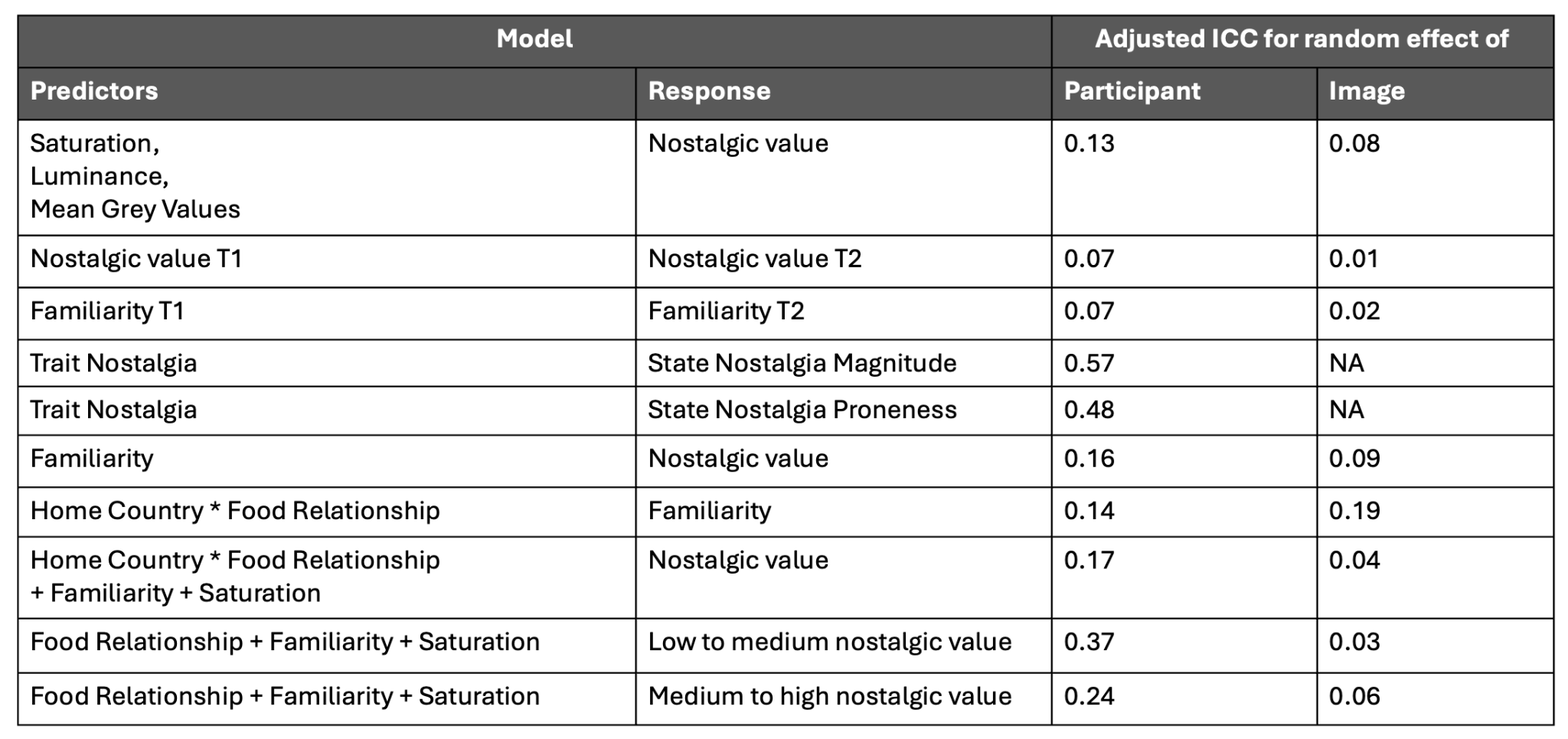


**Table E.** *Adjusted intraclass correlation coefficients for all random effects in each mixed-effects model reported in the results section.* Models are listed in the order in which they were reported.

**Appendix F: Spearman Correlations for Groups**

**
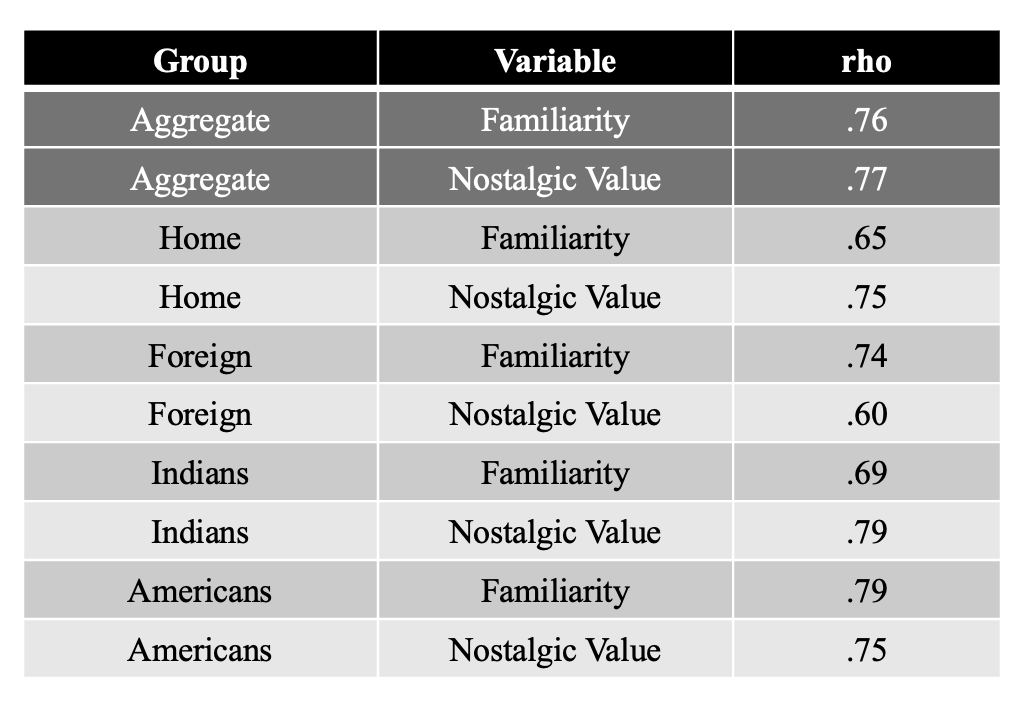
**

**Table F.** *rho values and significance levels for individuals’ first and second rating of the same item within groups.*
